# Clade III Synthases Add Cyclic and Linear Terpenoids to the *Psilocybe* Metabolome

**DOI:** 10.1002/cbic.202500167

**Published:** 2025-06-04

**Authors:** Nick Zschoche, Sebastian Schober, Karl Walther, Andrew R. Chadeayne, Markus Gressler, Stefan Bartram, Sarah E. O’Connor, Dirk Hoffmeister

**Affiliations:** ^1^ Pharmaceutical Microbiology Friedrich Schiller University Beutenbergstrasse 11a 07745 Jena Germany; ^2^ CaaMTech LLC 58 East Sunset Way Issaquah WA 98027 USA; ^3^ Department Natural Product Biosynthesis Max Planck Institute for Chemical Ecology Hans‐Knöll‐Strasse 8 07745 Jena Germany; ^4^ Pharmaceutical Microbiology Leibniz Institute for Natural Product Research and Infection Biology – Hans Knöll Institute Beutenbergstrasse 11a 07745 Jena Germany; ^5^ Cluster of Excellence Balance of the Microverse Friedrich‐Schiller‐Universität Jena Neugasse 23 07743 Jena Germany

**Keywords:** chromatography, enzymes, *psilocybe*, sesquiterpenes, terpene synthases

## Abstract

*Psilocybe* “magic mushrooms” are best known for their indolethylamine psilocybin, yet they encode enzymes for a much more diverse arsenal of small and potentially bioactive molecules. Herein, four *Psilocybe cubensis* clade III sesquiterpene synthases, CubB‐CubE, whose genes are differently expressed in fruiting bodies compared to vegetative mycelium are reported. CubB‐CubE were functionally characterized in vitro by product formation assays with heterologously produced enzymes and in vivo by transgene expression in *Aspergillus niger*, followed by extensive gas chromatography‐mass spectrometry analyzes. CubB was identified as a single product (3*R,*6*E*)‐(‐)*‐*nerolidol synthase. CubC is a multiproduct enzyme producing β‐caryophyllene, β‐elemene, α‐humulene, and β‐farnesene. CubD and CubE catalyze (near‐)exclusively sterpurene formation. *P. cubensis* young fruiting bodies and vegetative mycelium were analyzed for sesquiterpenes, which verified the presence of the CubB product α‐(3*R,*6*E*)‐(‐)*‐*nerolidol. As various *Psilocybe* species encode highly similar enzymes, this study contributes generally to the as‐yet little‐understood secondary metabolome of the genus.

## Graphical abstract

The *Psilocybe cubensis* terpene synthases, CubB‐CubE, were investigated. Product formation assays identified CubB as a (3*R*,6*E*)‐(‐)‐nerolidol or linalool synthase, whereas CubC is a multiproduct synthase catalyzing the formation of β‐caryophyllene and other linear and cyclic sesquiterpenes/‐terpenoids. CubD and CubE catalyze predominantly sterpurene formation. The results help to explore the true natural product diversity of this iconic mushroom genus.

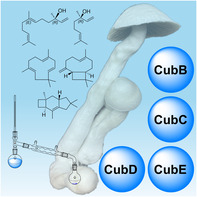

## 1 Introduction

Arguably, the most remarkable feature of so‐called “magic mushrooms” of the genus *Psilocybe* is the capacity to elicit psychedelic effects in humans. Historically, these effects were attributed solely to the presence of their eponymous natural product, psilocybin.^[^ ^1^ ^]^ This 4‐*O*‐phosphorylated indole alkaloid serves as a prodrug of the psychoactive compound, psilocin, which acts as a partial agonist of various serotonin receptors.^[^ ^2^ ^]^ Given the profoundly mind‐altering effects, it is little surprising that profiling the metabolites of the genus *Psilocybe* has traditionally focused on psilocybin and other isolated tryptamine alkaloids.^[^ ^3^ ^]^ Yet, increasing efforts in natural product chemistry to chart the often overlooked *Psilocybe* natural compound space revealed more bioactive nitrogen‐containing compounds, β‐carbolines and diketopiperazines in mycelia or carpophores.^[^ ^4^ ^]^ Still, more profound insight into the small‐molecule repertoire of *Psilocybe cubensis* and the genus *Psilocybe* generally is warranted.

Despite their controlled status in numerous countries, *P. cubensis* and other psilocybin‐containing species are home‐grown or collected and consumed for their psychedelic effects. Jurisdictional prohibitions of psilocybin in the United States and around the world have recently been lifted or relaxed, which may lead to an increased consumption of *Psilocybe* mushrooms. Severe yet reversible paralyses following consumption of magic mushrooms have occasionally been reported as early as 1973.^[^ ^5^ ^]^ Yet, these reported effects have never been systematically investigated and described in the literature. Hence, increased consumption may lead to a rising number of toxicological issues, elevating the unmet need for comprehensive knowledge on active mushroom molecules cooccurring with psilocybin. Furthermore, compounds other than psilocybin that are present in the mushrooms may interfere with its mode of action. This effect may, at least in part, explain a skeptically‐discussed so‐called “entourage effect”.^[^ ^6^ ^]^ This term refers to the theory that pure psilocybin purportedly elicits a different psychedelic response in the human body than psilocybin‐containing mushroom biomass. A recent study supports this notion as prolonged synaptic plasticity in mice was significantly more pronounced with *Psilocybe* extracts than with synthetic psilocybin.^[^ ^7^ ^]^

According to numerous genomic sequence data and chemical investigations of model organisms, the most diverse class of mushroom natural products, within and outside the genus *Psilocybe*, are by far the sesquiterpenes and terpenoids.^[^ ^4^, ^8^ ^]^

The structures of these volatile or nonvolatile compounds are linear or feature one or up to three carbon rings and frequently multiple stereocenters. Numerous terpenoids are bioactive and predestined to bind to and interfere with receptors.^[^ ^9^, ^10^ ^]^ Generally, the mode by which the universal sesquiterpene building block (2*E*,6*E*)‐farnesyldiphosphate (2*E*,6*E*‐FPP) is cyclized, and in some cases isomerized to (3*R*)‐nerolidyldiphosphate (NPP), by sesquiterpene synthases correlates with one of five phylogenetically distinct categories (clades I through V) these synthases fall into.^[^ ^11^ ^]^

**Figure 1:**
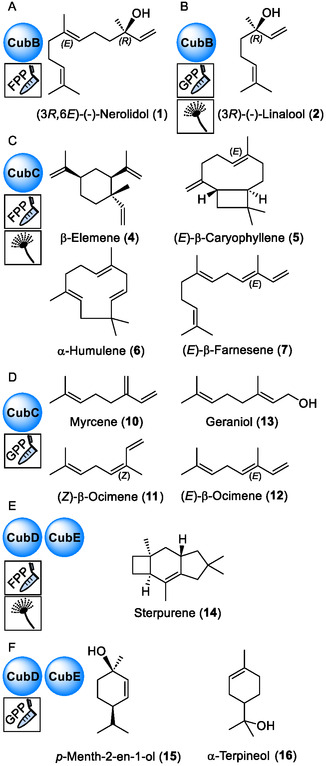
Chemical structures of *P. cubensis* terpene/terpenoid natural products. Pictograms symbolize in vitro reactions (test tube) or in vivo reconstitution of enzymatic activity in *A. niger* (mold symbols). A) Nerolidol formed by CubB in vitro with FPP as substrate. B) Linalool is the CubB product formed in vitro using GPP as substrate, or in the heterologous expression host *A. niger* tNZ07. C) Main products of CubC detected in vitro with FPP as substrate or in *A. niger* tNZ09. D) In vitro products of CubC with GPP as substrate. E) Sterpurene is formed with recombinant CubD and CubE with FPP as substrate in vitro or in *A. niger* tKFW01 and tKFW02, respectively. F) Products of CubD and CubE detected in vitro with GPP as substrate. The absolute configurations shown in panels C–F are exemplary representations.

Here, we report on the characterization of four *P. cubensis* clade III sesquiterpene synthases, hereafter referred to as CubB‐CubE. In other mushroom genera, clade III synthases are involved in the biosynthesis of highly bioactive compounds, such as the cytotoxic illudins and the melleolide antibiotics.^[8c,d]^ Based on in vitro product formation assays with recombinant enzymes and on reconstitution of their activities in vivo, their terpene/terpenoid products were identified. The results add (3*R,*6*E*)‐(‐)*‐*nerolidol, (*E*)‐β‐caryophyllene, β‐elemene, sterpurene (**Figure** **1**), and other sesquiterpenes to the secondary metabolome of the genus *Psilocybe.*

## 2 Results and Discussion

### 2.1 Clade III Terpene Synthase Genes

**Figure 2:**
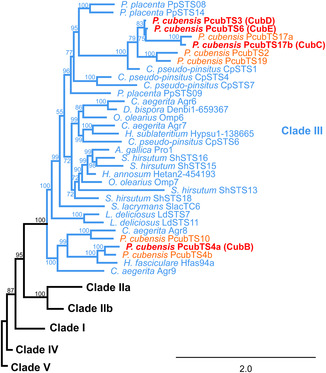
Phylogenetic tree of basidiomycete clade III sesquiterpene synthases and placement of *P. cubensis* putative (orange) and confirmed synthases (CubB‐CubE, red) within the cladogram. Details of the enzymes used to construct this tree are provided in Table S1, Supporting Information. Bootstrap values are indicated at each node. For reasons of clarity, clades I, IIa, IIb, IV, and V were collapsed.

*P. cubensis* encodes a total of 20 terpene synthases, nine thereof are members of clade III, as largest group. According to a previous study, five of these, preliminarily referred to as PcubTS2, PcubTS4b, PcubTS10, PcubTS17a, and PcubTS19, are transcriptionally (near‐)inactive both in vegetative mycelium and fruiting bodies.^[^ ^12^ ^]^ Hence, they were unlikely to contribute to the secondary metabolome and were not considered further. The same prior study recognized the genes coding for two sesquiterpene synthases (preliminarily designated PcubTS4a and PcubTS17b) of unknown function. The corresponding enzymes are, hereafter referred to as CubB and CubC, encoded by the most upregulated terpene synthase genes in fruiting bodies and representing two subclades within clade III. Conversely, genes for the two nearly identical synthases PcubTS03 and 06 (now referred to as CubD and CubE, respectively) were found downregulated in fruiting bodies, compared to vegetative mycelium. These findings were confirmed by quantitative real‐time polymerase chain reaction (qRT‐PCR), which indicated an 11.8‐fold upregulation of *cubB* and a sevenfold upregulation of *cubC* (Figure S1, Supporting Information), while *cubD* and *cubE* expression dropped to 0.15‐fold and 0.007‐fold in mushrooms. A phylogenetic analysis of basidiomycete clade III sesquiterpene synthases (**Figure** **2**, Table S1, Supporting Information) placed CubB next to *Hypholoma fasciculare* Hfas94a, a terpene synthase catalyzing mainly the formation of α‐humulene, and as a minor product, of (*E*)‐β‐caryophyllene (Figure 1).^[^ ^13^ ^]^ Together with Agr8 of *Cyclocybe aegerita*,^[^ ^14^ ^]^ a multiproduct synthase whose primary products are γ‐muurolene and β‐cadinene, the above‐mentioned synthases form an early diverging subclade within clade III. The closest relative to CubC‐CubE is the sterpurene synthase CpSTS1 of *Clitopilus pseudo‐pinsitus*.^[^ ^15^ ^]^ Interestingly, *cubD* and *cubE* share an 83.8% identity on the genetic level and are near‐identical on the amino acid level (95.6%, similarity 98.4%). Hence, both genes cluster in our phylogenetic study (Figure 2) next to each other and may have evolved by gene duplication.

The genes encoding CubB and CubC are each located in the proximity of a homologous gene for an almost identical synthase. For *cubB*, this ortholog, PcubTS4b, is located 1.2 kb away, whereas the *cubC* copy PcubTS17a is separated by 9.3 kb of DNA that harbors a transporter gene (Figure S2, Supporting Information). In the case of *cubB* and PcubTS4b, highly homologous genes (amino acid sequence identities between 78 and 90%, Table S2, Supporting Information) and (near‐)syntenic loci are conserved in other species as well, including *P. mexicana*, *P. serbica*, and *P*. *cyanescens* (Figure S2, Supporting Information).^[^ ^4^, ^16^, ^17^ ^]^ In the *P. azurescens* genome,^[4a]^ a more distantly related cluster of genes was detected (Figure S2, Supporting Information). In all of the above species, the *P*. *cubensis* genes coding for CubC and PcubTS17a do not have obvious homologs with identities >61% (Table S2, Supporting Information).

In the case of *cubD* and *cubE*, these two highly homologous genes are located on two distinct and clearly separated loci in *P. cubensis* (Figure S3, Supporting Information). A similar genetic situation was observed for their orthologs in other *Psilocybe* species (Figure S3, Supporting Information). In the immediate vicinity of *cubD,* other putative natural product biosynthesis genes were not determined, except one for *P. serbica*. Conversely, a near identical gene cluster was observed around *P. cubensis* *cubE* and its homologs in the investigated *Psilocybe* species. Indeed, *cubE* is surrounded by genes encoding Cu‐dependent oxidases, nicotinamide adenine dinucleotide ‐dependent reductases, and major facilitator superfamily‐type transporters.

Generally, clade III synthases cyclize the universal sesquiterpene building block (2*E*, 6*E*)‐farnesyldiphosphate (FPP) into the trans‐humulyl cation intermediate and further into various scaffolds.^[^ ^8^, ^18^ ^]^ Some sesquiterpene synthases show activity with (2*E*)‐geranyldiphosphate (GPP) as well.^[^ ^8^, ^19^ ^]^ Despite the predictable cyclization and the unambiguous phylogenetic placement, the actual product(s) of CubB‐CubE are unknown. Therefore, we produced these four enzymes individually in *Escherichia coli* from which the recombinantly produced enzymes were purified and subsequently characterized in vitro. Additionally, *Aspergillus niger* served as a host to gain in vivo evidence for sesquiterpene production.

### 2.2 Characterization of CubB in Vitro and in Vivo

*P. cubensis* CubB is encoded by a 1265 bp gene including four introns, which results in a 1035 bp reading frame in the mature mRNA and cDNA, respectively, encoding the 344 aa cyclase. The *cubB* cDNA was cloned to yield pET‐28a‐based expression plasmid pNZ08 that encodes an N‐terminally hexahistidine‐tagged enzyme version. This plasmid was used to transform *E. coli* KRX, and a 42.0 kDa protein (Figure S4, Supporting Information), was produced by the induced culture.

**Figure 3:**
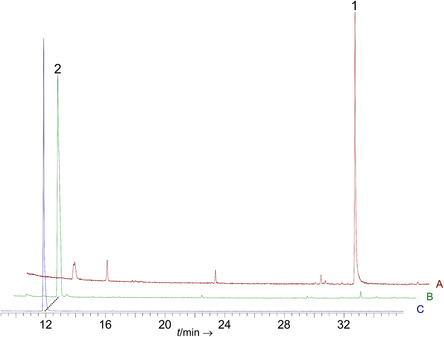
Gas chromatographic analysis of CubB activity assays. Chromatogram A) extract of the in vitro assay with FPP as substrate, yielding (3*R*,6*E*)‐(‐)‐nerolidol (**1**); B) mycelial extract of *A. niger* tNZ07 yielding linalool (**2**); and C) in vitro assay with GPP as substrate that led to the same enzymatic product.

CubB was purified by immobilized metal affinity chromatography (IMAC) and incubated with FPP as substrate for in vitro product formation assays. After extraction with *n*‐hexane, gas chromatography, coupled to electron impact mass spectrometry revealed a single product peak at *t*_R_ = 30.99 min (**Figure** **3**). Comparison of mass spectrum and retention index with databases (Table S3, Supporting Information)^[^ ^20^ ^]^ pointed to (3*R*,6*E*)‐(‐)‐nerolidol (Figure 1A). The absolute configuration was determined by chiral chromatography and comparison with a pure mixture of nerolidol diastereomeres (Figure S5, Supporting Information).^[^ ^21^ ^]^

Neither (3*R*,6*E*)‐(‐)‐nerolidol nor any other clade III sesquiterpene synthase product has been previously reported from a *Psilocybe* species. To confirm the results independently and in a cellular environment, CubB was heterologously produced in *A. niger*, transformed with plasmid pNZ13. The resulting transformant *A. niger* tNZ07 carried the *cubB* cDNA integrated into the genome (Figure S6, Supporting Information) and allowing for doxycycline‐dependent induction by an *Aspergillus* expression system.^[^ ^22^ ^]^ The *n*‐hexane extracts of mycelium, harvested from induced versus noninduced cultures of *A. niger* tNZ07, and from a control strain carrying the insertless vector (*A. niger* tPS01)^[^ ^23^ ^]^ and from the untransformed parental strain *A. niger* ATNT16Δ*pyrG*x24^[^ ^22^, ^24^ ^]^ were analyzed by gas chromatography‐mass spectrometry (GC‐MS) (Figure 3 and S7, Supporting Information). Starkly contrasting the results in vitro, a product peak at *t*_R_ = 11.93 min was found in vivo. A comparison with database entries and pure enantiomerically enriched (‐)‐linalool (ee = 87%), identified this peak after chiral separation^[^ ^21^ ^]^ as the linear monoterpenoid alcohol (3*R*)‐(‐)‐linalool (Figure 1B and S5, Table S4, Supporting Information), while the chromatogram did not indicate sesquiterpene/‐terpenoid formation.

There is precedent for fungal sesquiterpene synthases that yield monoterpenes/‐terpenoids when offered GPP, for example, in vitro work with *Coprinopsis cinerea* enzymes Cop4 and Cop6, which are clade II and clade IV synthases.^[8a]^ Furthermore, clade III sesquiterpene synthases, producing both nerolidol and linalool, were described from *Cyclocybe* species.^[^ ^25^ ^]^ To confirm monoterpene formation independently, we repeated the in vitro product formation assay with CubB and added GPP as substrate. GC‐MS analysis, as well as a second round of gas chromatography on a chiral column, identified (3*R*)‐(‐)‐linalool (Figure 1A) as the sole product (Table S3, Supporting Information).

To clarify the substrate preference, an in vitro substrate competition assay in the presence of equimolar concentrations of GPP and FPP was performed (50 μm each). The subsequent GC‐MS analysis (Figure S8, Supporting Information) indicated an approximately equal preference for these substrates as the observed product ratio was 53.02% (± 0.641) (*R*)‐(‐)‐linalool (**2**) and 46.98% (± 0.641) *trans*‐nerolidol (**1**). This result identifies CubB as a bifunctional mono‐/sesquiterpene synthase.

### 2.3 Characterization of CubC In Vitro and In Vivo

The second gene that encodes a clade III terpene synthase and which is highly upregulated in fruiting bodies is *cubC*. Of note, CubC (and likewise the subsequently described terpene synthases CubD and CubE) belong to the main subclade within clade III (Figure 2), as opposed to CubB, which falls into the earliest diverging branch of mushroom sesquiterpene synthases.

**Figure 4:**
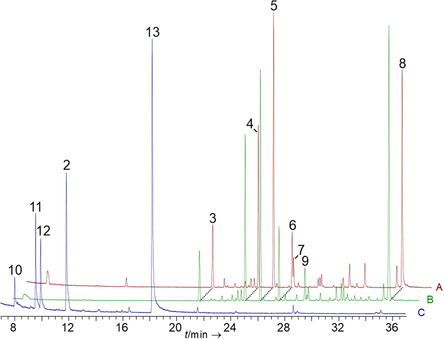
Gas chromatographic analysis of CubC‐catalyzed sesquiterpene formation. Assays or mycelia were extracted with *n*‐hexane. A) Extract of the in vitro product formation assay with FPP. B) Extract of a doxycycline‐induced culture of *A. niger* tNZ09. Chromatograms of controls are shown in Figure S9, Supporting Information. C) In vitro assay with GPP as substrate. Compound numbers: linalool (**2**), unidentified (**3**), β‐elemene (**4**), β‐caryophyllene (**5**), α‐humulene (**6**), β‐farnesene (**7**), unidentified (**8**), unidentified (**9**), myrcene (**10**), (*Z*)‐β‐ocimene (**11**), (*E*)‐β‐ocimene (**12**), and geraniol (**13**). The structures of the identified compounds are shown in Figure 1.

The *cubC* gene is disrupted by five introns, which results in a 1086 bp cDNA. Its product is a 361 aa terpene synthase with a predicted molecular mass of 40.9 kDa for the native, untagged protein. The investigation of CubC essentially followed the strategy described for CubB. First, hexahistidine‐tagged CubC was isolated from *E. coli* KRX, transformed with expression plasmid pNZ12 (Figure S4, Supporting Information), and purified by IMAC for subsequent in vitro product formation assays, run with FPP as substrate. Gas chromatographic analysis of the *n*‐hexane extracts and comparison of mass spectra and retention indices with the databases^[^ ^20^ ^]^ with pure compounds and with the plant essential oils of *Piper cubeba* and *Canarium* *luzonicum* identified CubC as a multi‐product synthase (**Figure** **4**, Table S5, Supporting Information). The main product (26.8% of the total peak area, *t*_R_ = 25.26 min) was (*E*)‐β‐caryophyllene (Figure 1B). Other products that each contributed >3% to the total peak area were β‐elemene (15.5% peak area, *t*_R_ = 24.13 min), α‐humulene (5.4% peak area, *t*_R_ = 26.64 min), (*E*)‐β‐farnesene (3.1% peak area, *t*_R_ = 26.74 min), as well as two unidentified products, compounds **3** and **8** (7.4 and 24.2% peak area, respectively) at *t*_R_ = 20.75 and 34.79 min. Very minor amounts of further sesquiterpenoids were present as well.

The in vitro assays were accompanied by in vivo work. *A. niger* was transformed with plasmid pNZ15 to introduce a doxycycline‐inducible copy of *cubC* cDNA in its genome, (Figure S9, Supporting Information). After induction, the terpene product spectrum of this transformant, *A. niger* tNZ09, was chromatographically investigated and found qualitatively and quantitatively similar to the in vitro‐generated sesquiterpene products (Figure 4, Table S6, Supporting Information). The unidentified compound **8** at *t*_R_ = 34.78 min was the dominant compound in vivo, followed by (*E*)‐β‐caryophyllene, (*E*)‐β‐elemene, and α‐humulene as the second to fourth most abundant product (Figure 1B). Dissimilar to the in vitro assays, (*E*)‐β‐farnesene was not found. Instead, an unidentified sesquiterpene (compound **9**) at *t*_R_ = 28.55 min and 3.3% of the total peak area was detected. As done for the CubB work, three controls were run in parallel, including *A.* *niger* tNZ09 uninduced, *A. niger* tPS01, carrying the insertless vector,^[^ ^23^ ^]^ and the uninoculated culture as control (Figure S10, Supporting Information).

For an initial characterization of **8**, the mass ion fragmentation pattern of the CubC in vitro assay with FPP was reanalyzed by GC‐MS. Three even fragments compatible with a possible bisabolene‐type structure (Figure S11, Supporting Information) were found. The chemical composition of the molecular ion peak (*m*/*z* 222.3%, C_15_H_26_O^·+^) and the fragments (*m*/*z* 207.10%, (C_14_H_23_O^+^, M^·+^‐ CH_3_), 204.10%, (C_15_H_24_^·+^, M^·+^‐ H_2_O), 180.23%, (C_12_H_20_O^·+^, M^·+^‐ C_3_H_6_, McLafferty rearrangement), and 68.53%, (C_5_H_8_^·+^, M^·+^‐ C_10_H_18_O, retro‐Diels–Alder reaction) was confirmed by GC‐high resolution mass spectrometry (HRMS). The loss of water to fragment *m*/*z* 204 may reflect a keto‐enol rearrangement of **8**, followed by an elimination. However, a bisabolene backbone is inconsistent with the established cyclization mode of clade III sesquiterpene cyclases, which is the 1,11‐cyclization of FPP. Rather, bisabolene formation requires 1,6‐cyclization familiar from clade IV cyclases.

Given the turnover of GPP by CubB and the dissimilar product spectra in vitro and in vivo, we repeated the CubC in vitro assays with this substrate. In the chromatographically analyzed extracts, the monoterpene alcohols (3*R*)‐ and (3*S*)‐linalool were found in a ratio of 3:2, as well as geraniol, myrcene, and (*Z*)‐ and (*E*)‐β‐ocimene (Figure 1D and 4, Table S7, Supporting Information).

### 2.4 Characterization of CubD and CubE In Vitro and In Vivo

Six introns are present in either gene, in each case leading to a 1092 bp cDNA and a 364 aa terpene synthase with a predicted mass of 41.6 kDa for the native proteins. N‐terminally hexahistidine fusion enzymes (Figure S12, Supporting Information) were purified from *E. coli* KRX that carried either plasmid pNZ07 (to express *cubD*) or pNZ09 (*cubE*). CubD and CubE in vitro product formation assays were also run with GPP and FPP as substrates in separate reactions, and subsequently analyzed by GC‐MS as described for the other synthases.

**Figure 5:**
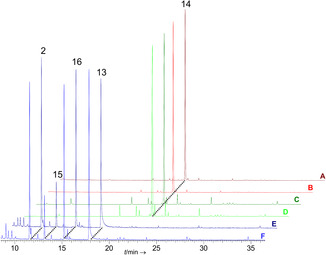
Gas chromatographic analysis of extracts of terpene formation by CubD and CubE. A) In vitro product formation assay with CubD and FPP. B) in vitro product formation assay with CubE and FPP. C) doxycycline‐induced culture of *A. niger* tKFW01 and D) of *A. niger* tKFW02. E) in vitro assay with CubD and GPP as substrate; and F) in vitro assay with CubE and GPP. Chromatograms of controls are shown in Figures S15 and S16. Compound numbers: linalool (**2**), geraniol (**13**), sterpurene (**14**), *para*‐menth‐2‐en‐1‐ol (**15**), and α‐terpineol (**16**).

With FPP as substrate, both enzymes catalyzed sterpurene formation (Figure 1E) as (near‐)exclusive product (*t*_R_ = 21.73 min, 92.28 and 92.15% of the peak area with CubD and CubE, respectively), and other mostly unidentified sesquiterpenes as very minor side products (**Figure** **5**, Table S8, Supporting Information). The reactions with GPP yielded qualitatively identical results for CubD and CubE as well, including linalool (**2**, Figure 1A), geraniol (**13**, Figure 1C), *para*‐menth‐2‐en‐1‐ol (**15**, Figure 1D), and α‐terpinenol (**16**), with very minor quantitative differences between the two enzymes (Table S9, Supporting Information). In a parallel approach, two genetically modified Aspergilli (*A. niger* tKFW01 and tKFW02, expressing *cubD* and *cubE* as transgenes, Figure S13 and S14) were created to determine the respective metabolite profile of CubD and CubE in vivo (Figure 5, Figure S15 and S16, Table S10, Supporting Information). After doxycycline‐induced transgene expression, either strain produced near‐exclusively sterpurene (**14**, Figure 1D), confirming our in vitro results.

### 2.5 Analysis of *P. cubensis* Mycelium and Fruiting Bodies

**Figure 6:**
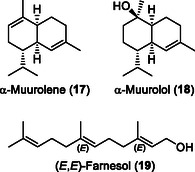
Chemical structure of α‐muurolene (**17**) and α‐muurolol (**18**) as main terpenoid compounds of *P. cubensis* mycelium, and (E,E)‐farnesol (**19**) found along nerolidol in young fruiting bodies.

Up to this point, the described sesquiterpenes/‐terpenoids were produced either by recombinantly made and purified *Psilocybe* enzymes in vitro, or in vivo in a heterologous host. To demonstrate that these enzymes play the expected enzymatic role in *P. cubensis*, we applied hydrodistillation of *P. cubensis* to verify that (some) CubB‐CubE products are part of this fungus's intrinsic secondary metabolome. First, vegetative mycelium was distilled and analyzed by GC‐MS. We detected CubC and CubD/CubE products β‐caryophyllene and sterpurene (Figure 1) in approximately equal amounts (4.5% and 5.2% of the total peak area of sesquiterpenes/‐terpenoids, respectively, Table S11, Supporting Information), along with β‐elemene and protoillud‐6‐ene as other typical products of clade III terpene cyclases, although the latter was not found in vitro or the heterologous system. The main products found after distillation were α‐muurolene (**17,** **Figure** **6**) and α‐muurolol (**18**, 35.6% and 28.1%, Table S11, Supporting Information). These compounds do very likely not result from the activity of the clade III cyclases CubB, CubC, CubD, or CubE as their biosynthesis requires 1,10‐cyclization of FPP, which is typical for clade I terpene cyclases and proceeds through an (*E*,*E*)‐germacradienyl cation.^[^ ^18^ ^]^

After hydrodistillation of young fruiting bodies, the major products detected chromatographically (Table S11, Supporting Information) included linear terpenoids (*E*,*E*)‐farnesol (**19,** Figure 6) and consistent with the strong upregulation of the *cubB* gene in fruiting bodies, the CubB product nerolidol (Figure 1A) with 43.2% and 37.2%, respectively.

Our in vitro assays, described earlier in this article, identified CubB as a bifunctional mono‐/sesquiterpene synthase. However, the exclusive linalool monoterpenoid formation in *A. niger* (i.e., the heterologous system) may simply reflect an insufficient supply with FPP, whereas this analysis of *P. cubensis* detected the sesquiterpenoid nerolidol as a major compound in fruiting bodies. Therefore, nerolidol formation appears to be the most plausible function of CubB under the physiological conditions of its source organism.

Our findings provide a comprehensive insight into the *P. cubensis* terpenome. Previous findings on *C. aegerita* showed that volatile production is a highly regulated process that depends on the developmental stage and age of the fruiting bodies.^[^ ^26^ ^]^ Therefore, further analytical work may likely be necessary to recognize the complete sesquiterpenome.

The presence of nerolidol in fruiting bodies matches the transcriptional profile of *cubB*, encoding the nerolidol synthase, which is upregulated in fruiting bodies. Likewise, sterpurene, made by CubD and CubE, was found in vegetative mycelia where the respective genes are transcribed. Conversely, the occurrence of β‐caryophyllene and β‐elemene, made by CubC, in mycelium seems incompatible with the upregulated transcription of *cubC* in fruiting bodies but could indicate a more complex temporal expression dynamic in the vegetative stage of *P. cubensis.*

## 3 Conclusion

While the alkaloid profile of the iconic mushroom *P.* *cubensis* is well‐studied, this mushroom also makes a wide variety of terpenoid natural products. To elucidate the metabolic capacity of *P.* *cubensis*, we mined the genome for uncharacterized terpene synthase genes. Here, we report the identification of CubB‐CubE, which we show to be substrate‐flexible clade III sesquiterpene synthases that accept FPP as well as GPP. Notably, these four enzymes belong to different evolutionary subclades and are dissimilar both in their products and the number of products, with CubB, CubD, and CubE being single‐product synthases, whereas CubC contributes multiple products to the repertoire of nitrogen‐free mushroom compounds.

Various *Psilocybe* species, beyond *P. cubensis*, encode highly similar enzymes. Therefore, we conclude that our findings on clade III terpene synthases apply to other species as well and contribute to a more global understanding of the secondary metabolome of the entire genus. This knowledge supports studies that address the open question to what extent the effects of pure psilocybin differ pharmacologically from the effects of psilocybin‐containing mushrooms on the human body.

## 4 Experimental Section

4.1

4.1.1

##### Materials and Microbial Strains

Chemicals, media ingredients, and solvents were purchased from Carbolution, Carl Roth, Merck, Sigma–Aldrich, and VWR. The reference mixture containing enriched (*R*)‐(‐)‐linalool and a mixture of racemic *cis*‐ and *trans*‐nerolidol for enantiomeric separation of the CubB products was from Aldrich. Oligonucleotides were synthesized by IDT Europe. *P. cubensis* strain FSU12409^[17a]^ was used for natural product and qRT‐PCR analysis. Mycelium was grown in liquid or on solid malt extract peptone  medium (15 g L^−1^ malt extract, 3 g L^−1^ peptone, for solid medium, and 18 g L^−1^ agar was added). Incubation was for 7 d at 25 °C, liquid cultures were shaken at 140 rpm. *P. cubensis* carpophores were produced as described.^[3c]^ For heterologous production of terpene synthases, *E. coli* KRX (Promega) was used. *A. niger* ATNT16Δ*pyrG*x24^[^ ^24^ ^]^ was used as host for terpene formation in vivo and grown on *Aspergillus* minimal medium (AMM), supplemented with 100 mm d‐glucose and 20 mm l‐glutamine, in flasks shaken at 140 rpm and 30 °C.

##### Bioinformatic Methods

The evolutionary relationship of basidiomycete terpene clade III synthases and the phylogenetic placement of CubB‐CubE was inferred using IQTree Web Server.^[^ ^27^ ^]^ Initially, the amino acid sequences of fungal sesquiterpene synthases of clades I–V were aligned using ClustalW2,^[^ ^28^ ^]^ implemented in the Geneious software version 10.2.4. Database entries for the clade III terpene synthase reference genes are given in Table S1, Supporting Information. The alignment was submitted to the IQTree Web Server, using the ultrafast bootstrap (UFBoot) feature^[^ ^29^ ^]^ and VT + F + G4 as identified best‐fit model. The bootstrap consensus tree with 141 taxa and 648 splits was inferred from 1000 replicates. Accuracy was additionally tested by the SH‐aLRT test.^[^ ^30^ ^]^ Bootstrap values are indicated at each node of the tree in case the SH‐aLRT was >80%.

##### Expression Analysis by qRT‐PCR

The biomass of *P. cubensis* (mycelium or carpophores) was ground under liquid nitrogen into a powder. *P. cubensis* gDNA was isolated following an established protocol^[^ ^31^ ^]^ and used to determine oligonucleotide primer efficiencies. RNA was isolated using the Promega SV Total RNA Isolation kit and subsequent treatment with Baseline‐Zero (Lucigen) to remove residual DNA. To produce cDNA for expression analysis and cloning of expression vectors (below), RNA (1 μg) was reverse transcribed with the RevertAid RT kit (Thermo) and an anchored oligo‐(dT)_18_ primer. The qRT‐PCRs were set up in a total volume of 20 μL, using 50 ng DNA template, 1 μm primer (each), and the EvaGreen qPCR mix (Bio&Sell). Sequences, target genes, and efficiencies of oligonucleotides are given in Table S12, Supporting Information. Thermal cycling parameters were: initial hold at 95 °C for 15 min, 40 cycles of 95 °C for 15 s, 60 °C for 20 s, and 72 °C for 20 s. Three biological and three technical replicates each were run. Genes encoding glyceraldehyde‐3‐phosphate dehydrogenase (*gpdA*), actin (*actA*), both as internal housekeeping reference genes, and the established carpophore‐specific marker gene (*mtdA*)^[^ ^32^ ^]^ served as controls. Gene expression levels were determined as described.^[^ ^33^ ^]^

##### Construction of Expression Plasmids

To express terpene synthase genes in *E. coli*, plasmids pNZ08, pNZ12, pNZ07, and pNZ09 were created based on the vector pET‐28a, carrying *cubB*, *cubC*, *cubD,* and *cubE,* respectively. The inserts were amplified by PCR in a total volume of 50 μL and the buffer supplied with the polymerase. The reactions contained 2 μm MgCl_2_, 200 μm each deoxynucleoside triphosphate, 10 μm each primer (oNZ15/oNZ16 to amplify *cubB*, oNZ23/oNZ24 for *cubC*, oNZ13/oNZ14 for *cubD*, and oNZ17/oNZ18 for *cubE* (Table S13, Supporting Information), template DNA (cDNA from the previous qRT‐PCR), and 1 U Phusion high‐fidelity DNA polymerase. The thermal cycling protocol was 95 °C, 2 min initial hold, followed by 31 cycles of 95 °C, 20 s, 60 °C, 20 s, 72 °C, 60 s, and a final hold at 72 °C for 10 min. PCR products were sequenced to ensure proper amplification and purified on agarose gels according to the manufacturer's instructions (Promega). The vector pET‐28a was linearized by simultaneous restriction with *Nhe*I and *Xho*I (to insert *cubB* or *cubC*), *Bam*HI and *Xho*I (*cubD*), and with *Nhe*I and *Not*I (*cubE*). Expression plasmids were created using the NEBuilder kit (NEB) following the manufacturer's instructions.

For transgene expression in *A. niger*, the vector pPS01^[^ ^23^ ^]^ was used to create plasmids pNZ13 (*cubB*), pNZ15 (*cubC*), pKFW03 (*cubD*), and pKFW04 (*cubE*). These plasmids confer uracil prototrophy in the uracil‐auxotrophic host. Vector pPS01 was linearized with *Spe*I and *Pac*I to prepare it for assembly. Inserts were prepared by PCR and the conditions described above, but using oligonucleotide pairs oNZ11/12 (*cubB*), oNZ03/04 (*cubC*), oKFW06/07 (*cubD*), and oKFW08/09 (*cubE*). Oligonucleotide sequences are shown in Table S14, Supporting Information.

##### Heterologous Enzyme Production

*N*‐terminally hexahistidine‐tagged enzymes CubB‐CubE were produced in *E. coli* KRX, transformed with pNZ08 (CubB), pNZ12 (CubC), pNZ07 (CubD), or pNZ09 (CubE), and grown in lysogeny broth medium, amended with 50 μg mL^−1^ kanamycin. The protein was purified as previously described,^[^ ^33^ ^]^ concentrated using a centrifugal filter (Amicon Ultra‐15, Merck, 10 kDa cutoff) and eluted with reaction buffer (50 mm TRIS, pH 8, and 10 mm MgCl_2_). Protein concentrations were measured with the Pierce BCA‐Protein Assay Kit (Thermo) at a wavelength of *λ* = 562 nm.

##### In vitro Product Formation Assays

Reactions were carried out in triplicates and with 1 μm TRIS‐buffered enzyme CubB‐CubE, and 50 μm (2*E*,6*E*)‐farnesyldiphosphate or (2*E*)‐geranyldiphosphate (Merck), or both substrates for the CubB competition assay, in a volume of 500 μL and at 35 °C for 30 min. The reactions were extracted with 200 μL *n*‐hexane, centrifuged, and filtered. Subsequently, 1 μL of the respective samples were used for GC‐MS analysis. As a control, a reaction with heat‐treated enzyme was run in parallel.

##### Transformation of A. niger and Transgene Expression

*A. niger* ATNT16Δ*pyrG*x24 protoplasts were prepared and transformed as described.^[22a]^ To select for the successful integration of expression plasmids, cells were grown on uracil‐free medium. Full‐length integration of *cubB*, *cubC*, *cubD*, or *cubE* cDNA in the transformants was verified by PCR. The reactions included oligonucleotides oMG360 as forward primer and oNZ26 (for *cubB*) and oNZ30 (*cubC*) as reverse primers. To detect *cubD* and *cubE*, forward primer oMG370 and reverse primers oKFW08 (*cubD*) or oKFW11 (*cubE*) were used (Table S15, Supporting Information), cDNA as template, and 2.5 U Taq polymerase. The thermal cycling protocol applied to construct expression plasmids (mentioned above) was used here as well. Verified transformants *A*. *niger* tNZ07, tNZ09, tKFW01, and tKFW02 (Figure S6, S9, S13, and S14) were selected and grown in 100 mL liquid AMM (20 g L^−1^ peptone, 20 g L^−1^ d‐glucose, and 10 g L^−1^ yeast extract) for 24 h at 30 °C, shaking at 180 rpm. After 24 h of growth, transgene expression was induced by adding 30 μg mL^−1^ doxycycline, and cultivation was continued for another 48 h. *A. niger* tPS01^[^ ^23^ ^]^ carrying the insertless vector pPS01 (negative control) and uninduced cultures of *A*. *niger* tNZ07, tNZ09, tKFW01, and tKFW02 served as controls.

##### Extraction of Aspergillus Biomass

The *A. niger* cultures were passed through Miracloth to separate the biomass from the broth for subsequent chromatographic analysis. The mycelium was rinsed with water to remove residual broth, blotted dry, shock‐frozen in liquid nitrogen, and ground with a mortar and pestle. The *n*‐hexane was added to the pulverized mycelium (500 μL per 1 g biomass), vigorously shaken, centrifuged, and subsequently used for GC‐MS (below).

##### Analysis of Terpenes in P. cubensis Mycelium and Fruiting Bodies

Vegetative mycelium (80 g fresh weight) for hydrodistillation was prepared from a liquid culture by filtration, washed with deionized water, and transferred to 500 mL round‐bottom flasks. Young *P. cubensis* carpophores (216 g fresh weight) produced in indoor cultures were quick‐frozen in liquid nitrogen. Subsequently, they were thawed, sliced, and transferred to 500 mL round‐bottom flasks. The samples were overlaid with 250 mL deionized water, amended with 500 μL silicon‐containing defoamer (Carl Roth). The essential oil was determined in a closed hydrodestillation apparatus. Distillation proceeded for 4 h at a constant flow velocity of 2 mL min^−1^. The essential oils were collected in 500 μL *n*‐hexane and subsequently subjected to gas chromatography.

##### Gas Chromatography and Mass Spectrometry

The products of the reactions with CubB‐CubE were analyzed on an ISQ GC‐MS‐System (Thermo Fisher Scientific), equipped with a ZB‐5MS‐column and inactive guard column (30 + 10 m × 0.25 mm × 0.25 μm, Phenomenex), essentially using the described parameters.^[^ ^34^ ^]^ Briefly, the temperature was increased by 3 °C min^−1^ from 60 to 260 °C. Helium was the carrier gas (1 mL min^−1^ flow) with splitless injection or in split‐mode (1:10). The injector temperature was maintained at 220 °C. Electron impact ionization was performed at 70 eV, the ion source was kept at 250 °C. The products of CubB with GPP and FPP are (3*R*)‐(‐)‐linalool and (3*R*,6*E*)‐(‐)‐nerolidol, respectively. Enantiomeric separation was accomplished on a hydrodex‐β‐3 P chiral GC‐column (Macherey‐Nagel), at a flow rate of 1 mL min^−1^ He and a heating rate of 1 °C min^−1^ from 50 to 150 °C. The order of elution of enantiomers followed published results.^[^ ^21^ ^]^ All spectra were measured in positive ionization mode. To deconvolute the data and to calculate the retention index,^[^ ^35^ ^]^ MassFinder (Hochmuth Scientific Consulting, Hamburg, Germany) was used. GC‐HRMS measurements were carried out on a Trace 1310 series gas chromatograph with a split/splitless injector coupled to a Q‐Exactive‐GC Orbitrap mass spectrometer (Thermo Fisher Scientific). Separations were obtained with a fused silica capillary column (ZB SemiVolatiles, 30 mm × 25 mm, 0.25 μm + 10 m Zebron guard column, Phenomenex) using at the same conditions as above. Resolution of the Orbitrap was set to 120 000 at 200 Da. GC‐MS results were analyzed using Xcalibur v3.1.66.10 software (Thermo Fisher, Waltham, MA, USA). Enzymatic products of CubB‐CubE were identified by comparing their mass spectra and retention indices with databases and literature.^[^ ^20^ ^]^ Additionally, retention times and mass spectra were compared with gas chromatographic runs, applying identical conditions, of pure compounds or essential oils of *P. cubeba* berries (Cubeb oil) and *C.* *luzonicum* resin (Elemi oil).^[^ ^36^, ^37^ ^]^

##### Statistical Analysis

qRT‐PCR results (Figure S1, Supporting Information) are based on triplicate runs (*n* = 3). The error bars indicate the standard deviation. qRT‐PCR data were collected and processed using qPCRsoft 4.0 software (AnalytikJena).

## Conflict of Interest

The authors declare no conflict of interest.

## Supporting information

Supplementary Material

## Data Availability

The data that support the findings of this study are available in the supplementary material of this article.
