# Supplementary material for: Clade III Synthases Add Cyclic and Linear Terpenoids to the Psilocybe Metabolome
Source: Chembiochem. 2025 Jun 4;26(13):e202500167. doi: 10.1002/cbic.202500167 (PMC12247026; doi:10.1002/cbic.202500167)
Supplement: Supplementary file 1 — Supplementary Material [file CBIC-26-e202500167-s001.pdf]

Table of Contents

Figure S1. Transcription analysis of *cubB* and *cubC* by qRT-PCR 2
Figure S2. Genetic map of terpene synthase genes *cubB*, *cubC* and adjacent genes 3
Figure S3. Genetic map of terpene synthase genes *cubD, cube* and adjacent genes 4
Figure S4. SDS polyacrylamide gel electrophoresis of purified His_6-tagged CubB and CubC. 5
Figure S5. Enantiomeric separation of the CubB products 6
Figure S6. PCR analysis of *cubB* transgene integration in *Aspergillus niger* tNZ07 7
Figure S7. CubB-catalyzed terpene formation in *Aspergillus niger* tNZ07 8
Figure S8. *In vitro* substrate competition assay with CubB as well as GPP and FPP 9
Figure S9. PCR analysis of *cubC* transgene integration in *Aspergillus niger* tNZ09 10
Figure S10. CubC-catalyzed terpene formation in *Aspergillus niger* tNZ09 11
Figure S11. GC-MS/MS spectra to identify compound **8** 12
Figure S12. SDS polyacrylamide gel electrophoresis of purified His_6-tagged CubD and CubE. 13
Figure S13. PCR analysis of *cubD* transgene integration in *Aspergillus niger* tKFW01 14
Figure S14. PCR analysis of *cubE* transgene integration in *Aspergillus niger* tKFW02 15
Figure S15. CubD-catalyzed terpene formation in *Aspergillus niger* tKFW01 16
Figure S16. CubE-catalyzed terpene formation in *Aspergillus niger* tKFW02 17

Table S1. Amino acid sequences of terpene synthases used for phylogenetic analyses 18
Table S2. Predicted terpene synthases encoded by various *Psilocybe* species 20
Table S3. Mono- and sesquiterpenes and -terpenoids produced by CubB *in vitro* 21
Table S4. Identified product in extracts of *Aspergillus niger* tNZ07 21
Table S5. Sesquiterpenes produced by CubC *in vitro* 22
Table S6. Identified products in extracts of *Aspergillus niger* tNZ09 23
Table S7. Monoterpenes and -terpenoids produced by CubC *in vitro* 24
Table S8. Sesquiterpenes and -terpenoids produced by CubD and CubE *in vitro* 25
Table S9. Monoterpenes and -terpenoids produced by CubD and CubE *in vitro* 26
Table S10. Identified products in extracts of *Aspergillus niger* tKFW01 and tKFW02 27
Table S11. Identified products after hydrodistillation of *Psilocybe cubensis* fruiting bodies or mycelium 28
Table S12. Oligonucleotides for qRT-PCR 30
Table S13. Oligonucleotides to construct *Escherichia coli* expression plasmids 30
Table S14. Oligonucleotides to construct *Aspergillus niger* expression plasmids 31
Table S15. Oligonucleotides for diagnostic PCR to verify transgene integration 31

References. 32

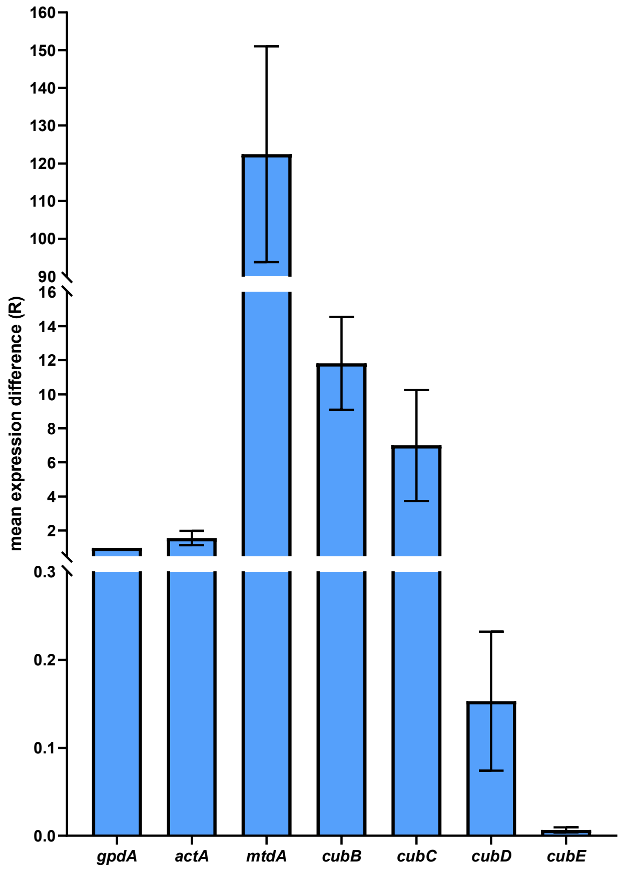

Figure S1. Expression of *Psilocybe cubensis cubB-cubE.* qRT-PCR was used to compare expression in fruiting bodies with mycelium. The mean expression difference was normalized using the housekeeping gene, *gpdA*, which was set to one. The primers to amplify *cubB* and *cubC* were designed to anneal to up- and downstream exon sequences flanking intron 4 and 5, respectively. For *cubD* and *cubE*, the amplicon included the first exon in either gene. Accurately spliced mRNA leads to amplicons of 103 bp (*cubB*), 145 bp (*cubC*), 83 bp (*cubD*), and 89 bp (*cubE*). The genes *actA* (encoding actin) was included as an additional negative control. The gene *mtdA* (encoding a putative peptide transporter) is an established carpophore-specific marker and was included as positive control for the developmental transition from mycelium to carpophores.[32,33] The bars indicate the standard deviation. Values above and below 1 indicate upregulation and downregulation, respectively, in the fruiting body, compared to the mycelium. PCRs were run in triplicate (n = 3).

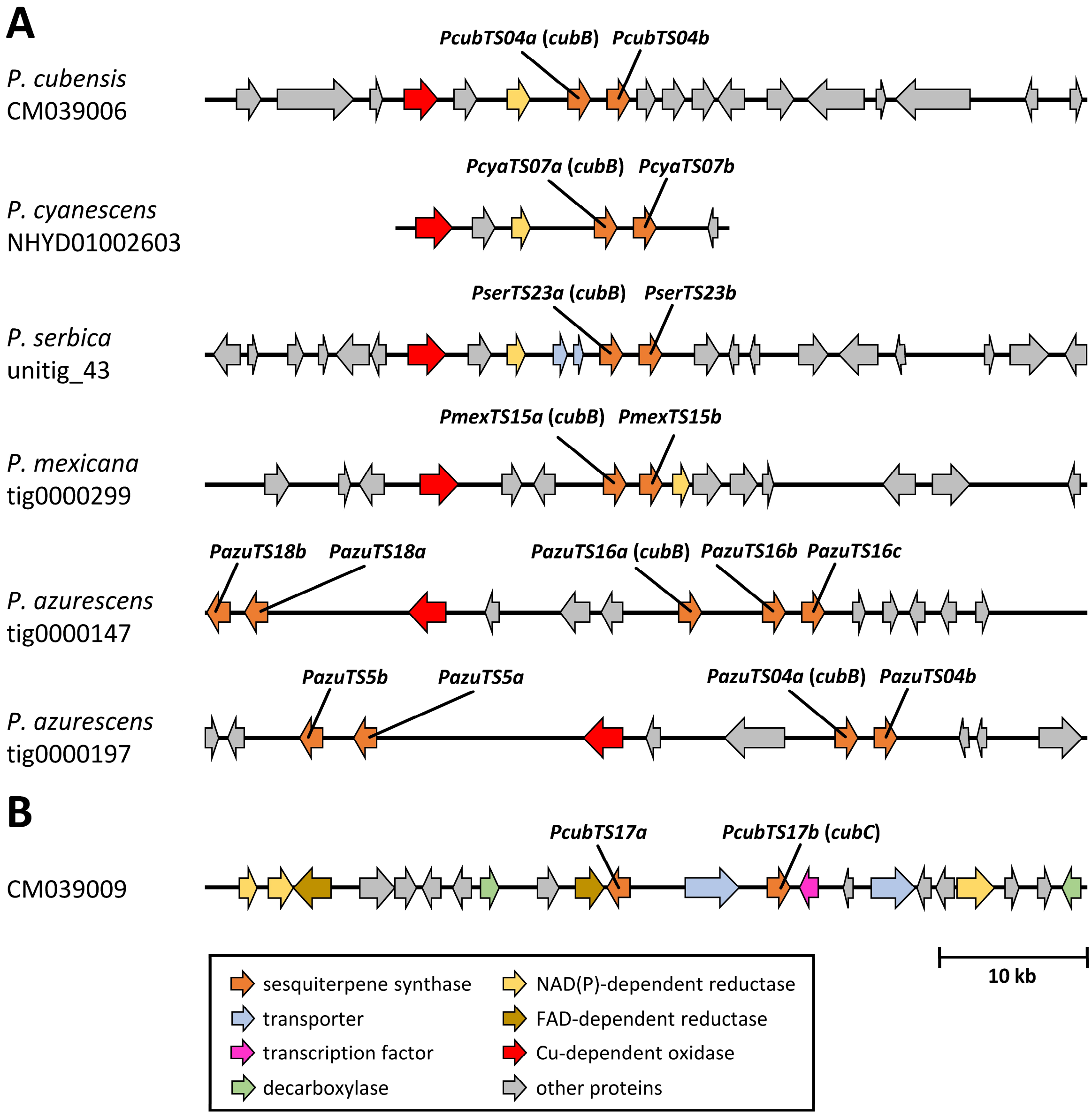

Figure S2. Genetic map of *cubB* and *cubC* and adjacent genes.

A) The combination of *cubB* and genes putatively encoding PcTS4b as well as a copper-dependent oxidase and an NAD(P)H-dependent reductase in *P. cubensis* was found (near-)syntenically in the genomes of *P. cyanescens*, *P. serbica*, and *P. mexicana*[4a,17a,17b] as well which may suggest an ancient evolutionary origin prior to the divergence of the *mexicanae* and *cubensae* clades within the *Psilocybe* genus.[38] Two distantly related clusters of genes are present in the *P. azurescens* genome as well.[4a,17a,17b] Please refer to Table S2 for sequence similarities of terpene synthases,

B) Genetic situation around *cubC* and the gene PcTS17a. The arrows indicate the transcriptional direction of the genes, introns are not shown. The genes coding for oxidases, reductases, decarboxylases, for the transcription factor and transporter (please refer to the color code) may not be related to terpene/terpenoid production by *P. cubensis*. The contig numbers refer to the published assemblies.[39]

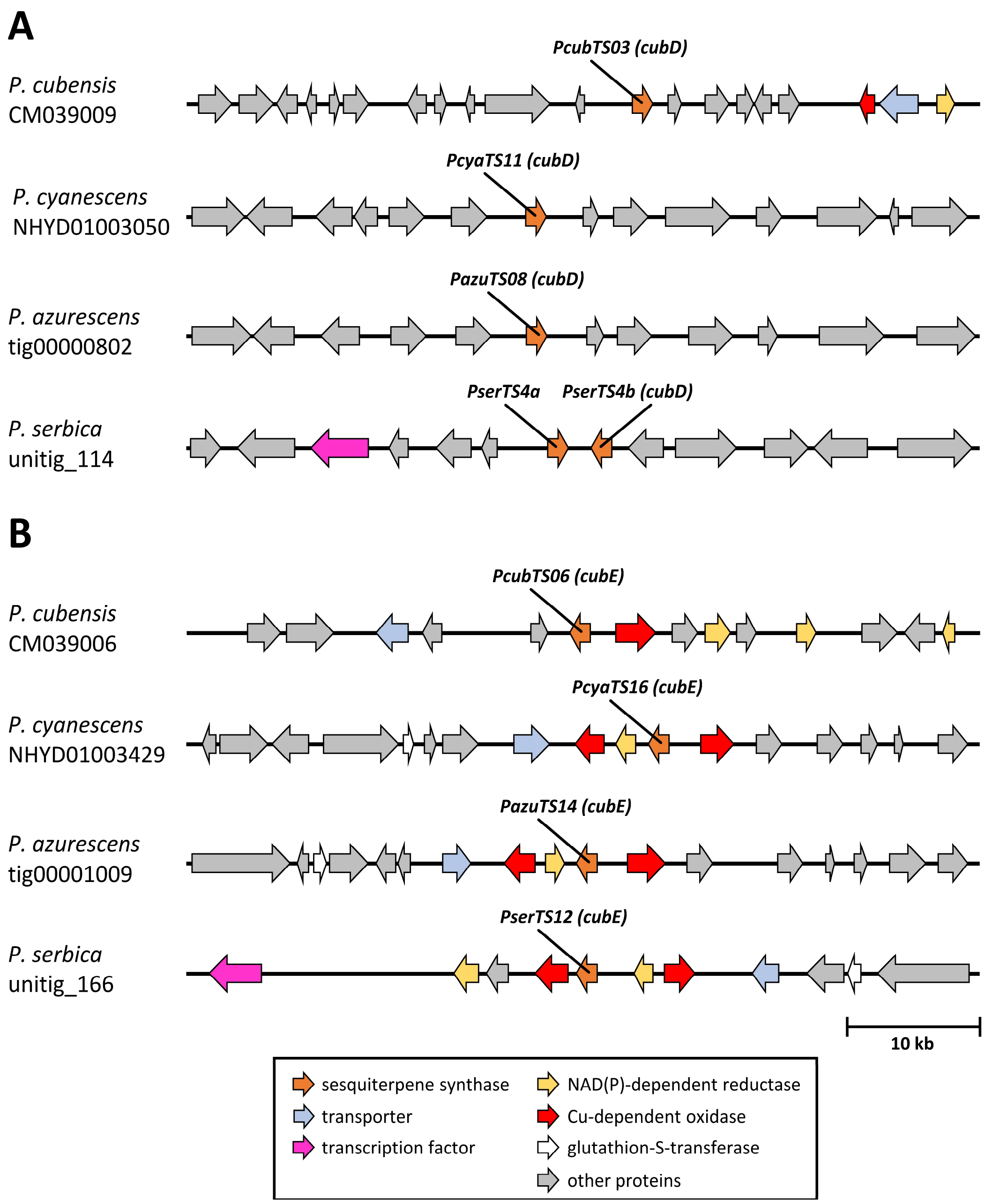

Figure S3. A) The genetic situation around *cubD* in *P. cubensis* and homologs in three other well-characterized *Psilocybe* species implies a solitary localization. A cluster of biosynthetic genes was not apparent in any of the genomes. However, in *P. serbica*, *cubD* is located adjacent to an additional sesquiterpene cyclase gene unrelated to *cubD*. Please refer to Table S2 for sequence similarities of terpene synthases.

B) *cubE* and homologs, and adjacent genes. The latter encode one (*P. cubensis*) or two (other species) copper-dependent oxidases (red), one (*P. cyanescens* and *P. azurescens*), two (*P. serbica*) or three (*P. cubensis*) NAD(P)H-dependent reductases (yellow) and one transporter of the major facilitator superfamily or the ATP-binding cassette transporter-type (light blue). The arrows indicate the transcriptional direction of the genes, introns are not shown. The genes coding for oxidases, reductases, transcription factors and transporters (please refer to the color code) may not be related to terpenoid production by *P. cubensis*. The contig numbers refer to the published assemblies.[39]

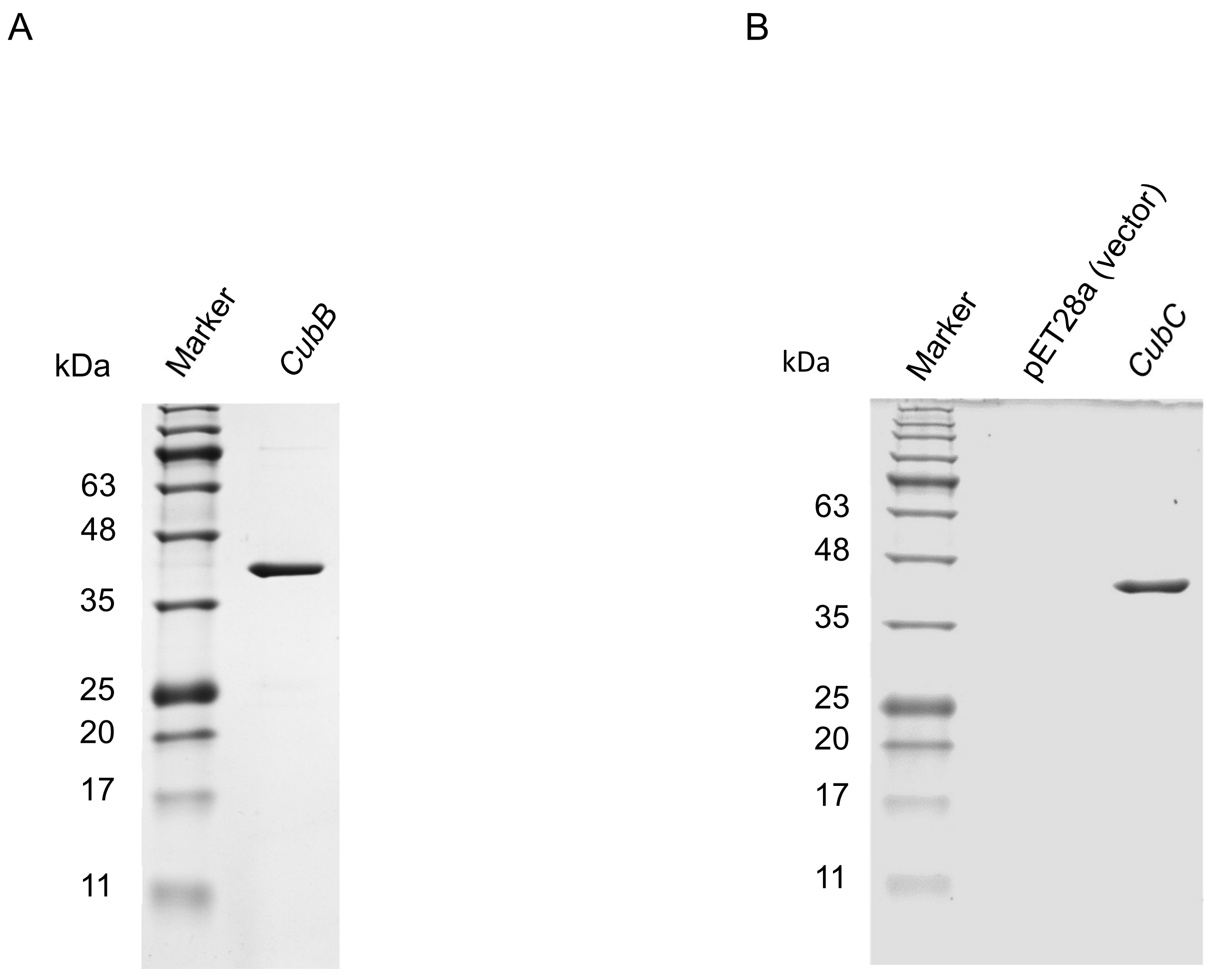

Figure S4. SDS polyacrylamide gel electrophoresis of purified His$_6$-tagged *Psilocybe cubensis* CubB (panel A) and CubC (panel B). The calculated masses of hexahistidin-tagged CubB and CubC are 42.0 and 43.4 kDa, respectively. An extract of *E. coli*, transformed with the empty expression vector pET28a, is shown as negative control. Protein standard: Blue Eye prestained marker (Jena Bioscience).

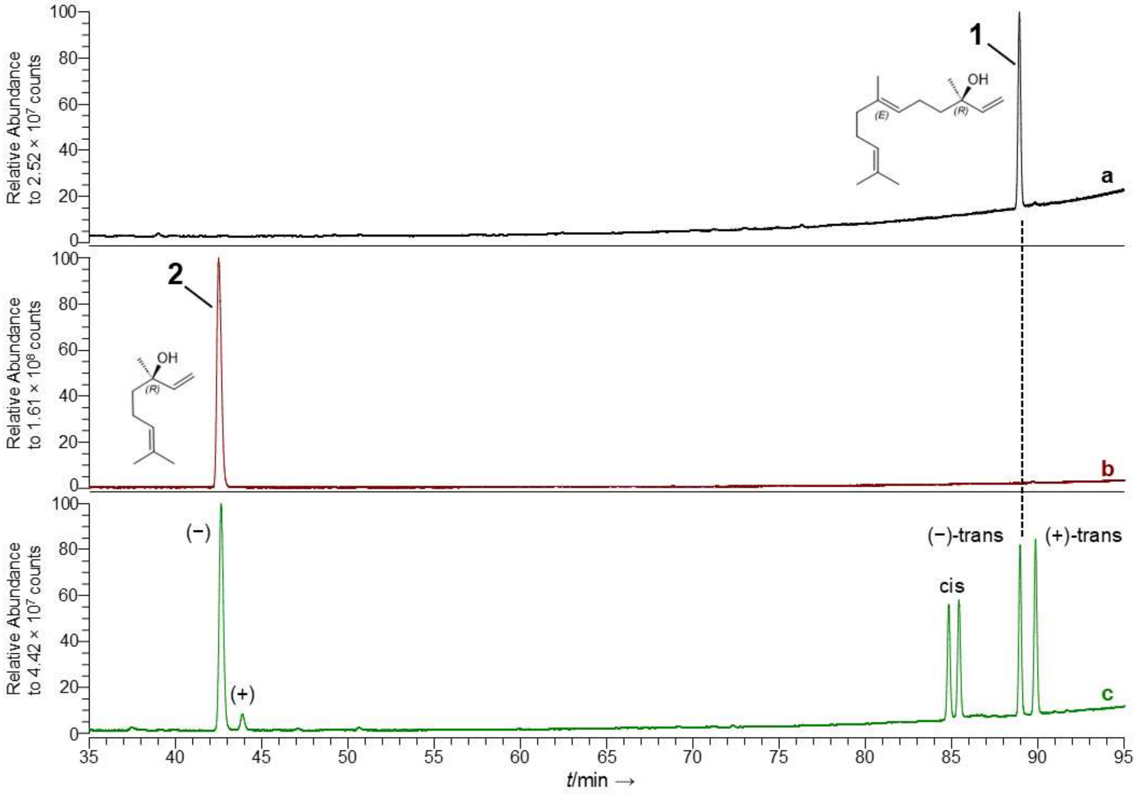

Figure S5. Enantiomeric gas chromatographic separation of the CubB products in the presence of GPP or FPP as substrate. Top chromatogram: *in vitro* reaction of CubB with FPP as substrate; center chromatogram: CubB reaction with GPP; bottom chromatogram: reference mixture that contained enriched (*R*)-(-)-linalool (**2**) (ee = 87%) and a mixture of racemic *cis*- and *trans*-nerolidol (**1**) in n-hexane.

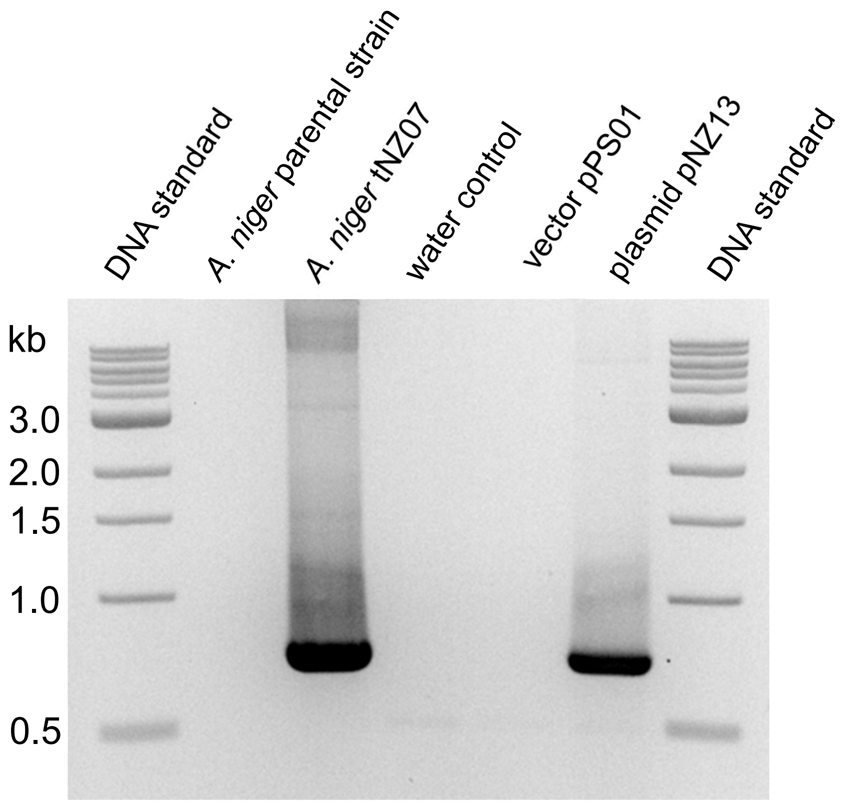

Figure S6. Agarose gel electrophoresis of PCRs to verify integration of the *cubB* expression construct pNZ13 in the *Aspergillus niger* genome. The integration yielded producer strain *A. niger* tNZ07. Negative controls i) with genomic DNA of the untransformed parental strain *A. niger* ATNT16ΔpyrGx24, ii) water control without template DNA, iii) a reaction with empty vector DNA (pPS01), and a positive control (plasmid pNZ13) are shown as well.

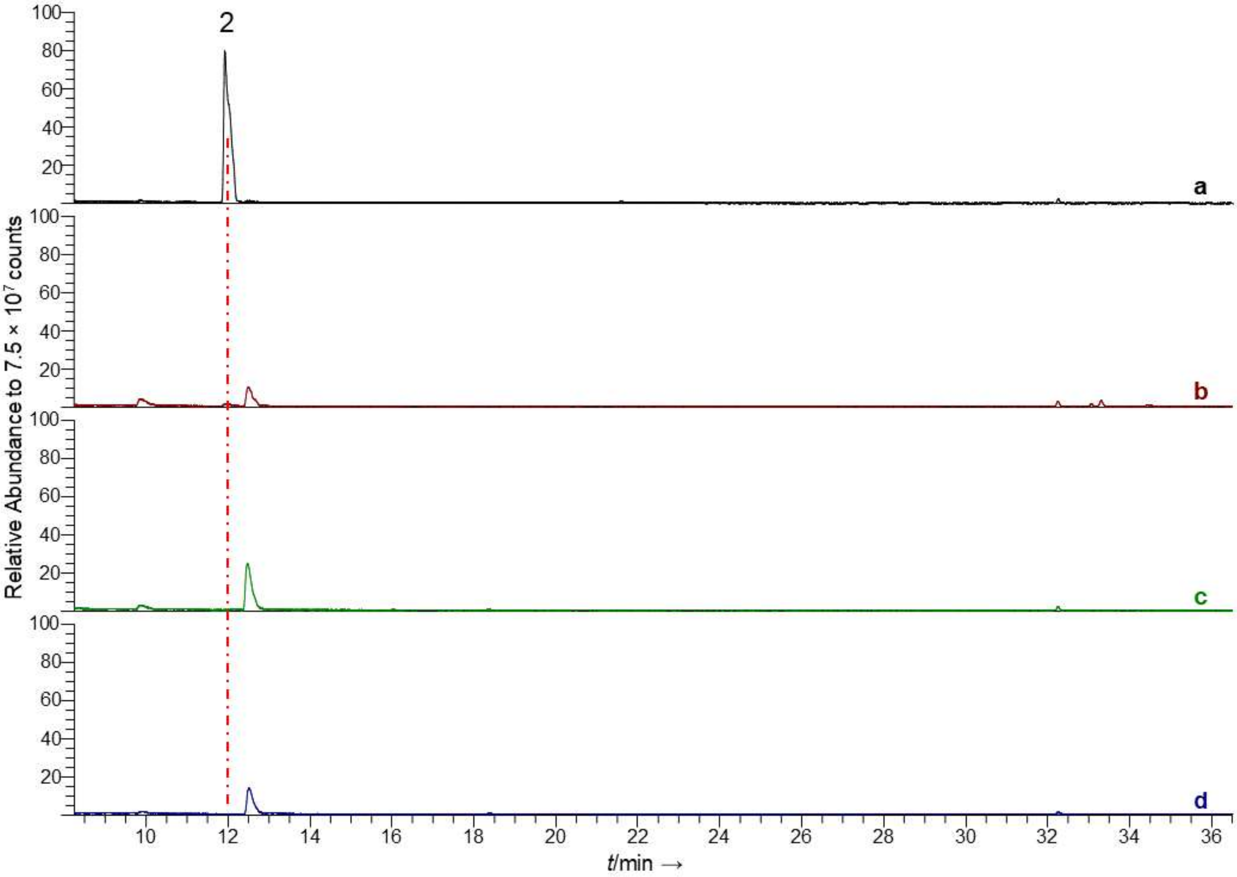

Figure S7. Gas chromatographic analysis of CubB-catalyzed terpenoid formation in *Aspergillus niger* tNZ07. Shown are *n*-hexane extracts of an (a) doxycycline-induced and (b) non-induced culture of *A. niger* tNZ07. Chromatograms c and d represent extracts of induced cultures of the empty vector control strain, *A. niger* tPS01, and the parental strain *A. niger* ATNT16ΔpyrGx24. Compound numbering: (*R*)-(-)-linalool (**2**).

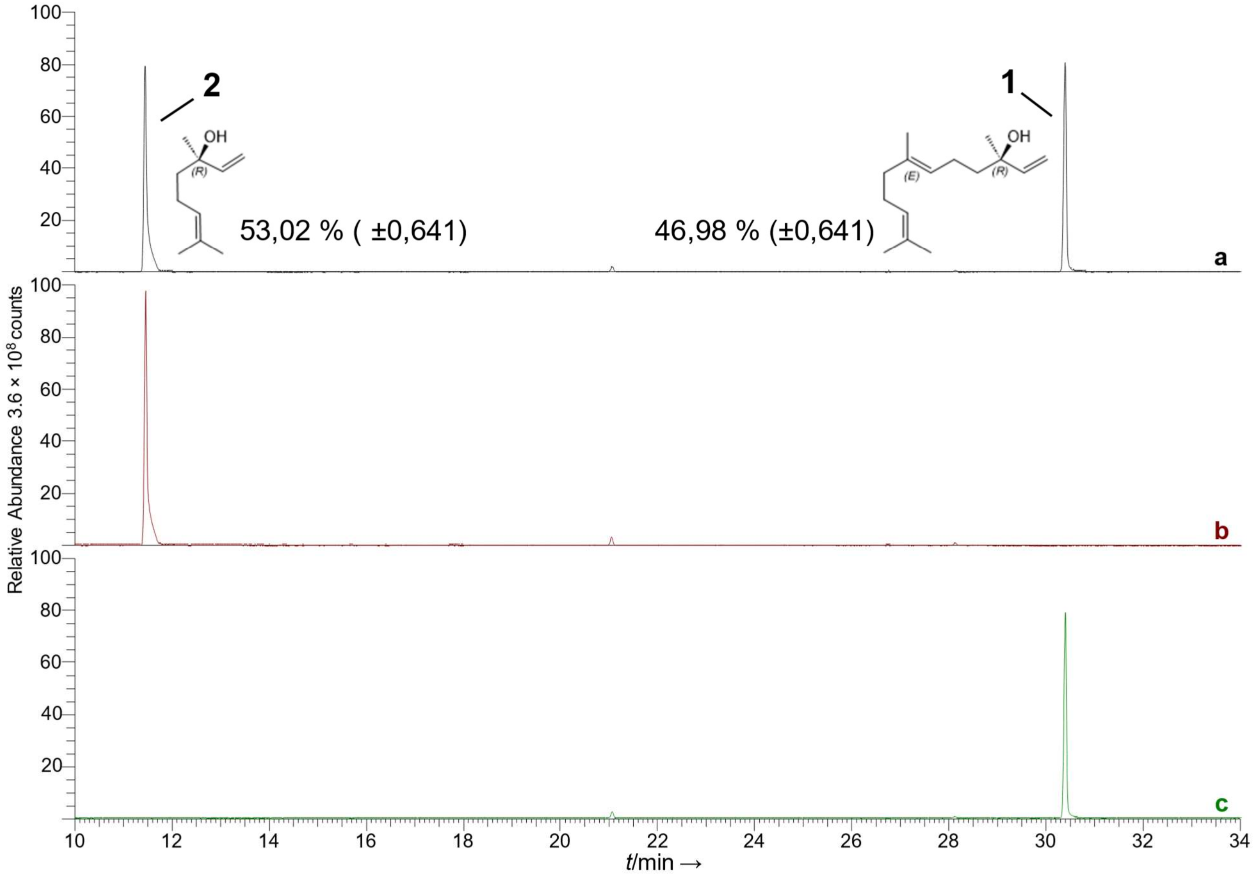

Figure S8. In vitro substrate competition assay with CubB as well as GPP and FPP. a) *in vitro* reaction of CubB in the presence of equimolar concentrations (50 µM each) of GPP and FPP, leading to peak areas of products (*R*)-(-)-linalool (**2**) 53,02% (±0,641) and *trans*-nerolidol (**1**) 46,98% (±0,641); b) CubB reaction with GPP as single substrate; c) CubB assay with FPP. Assays were run in triplicates.

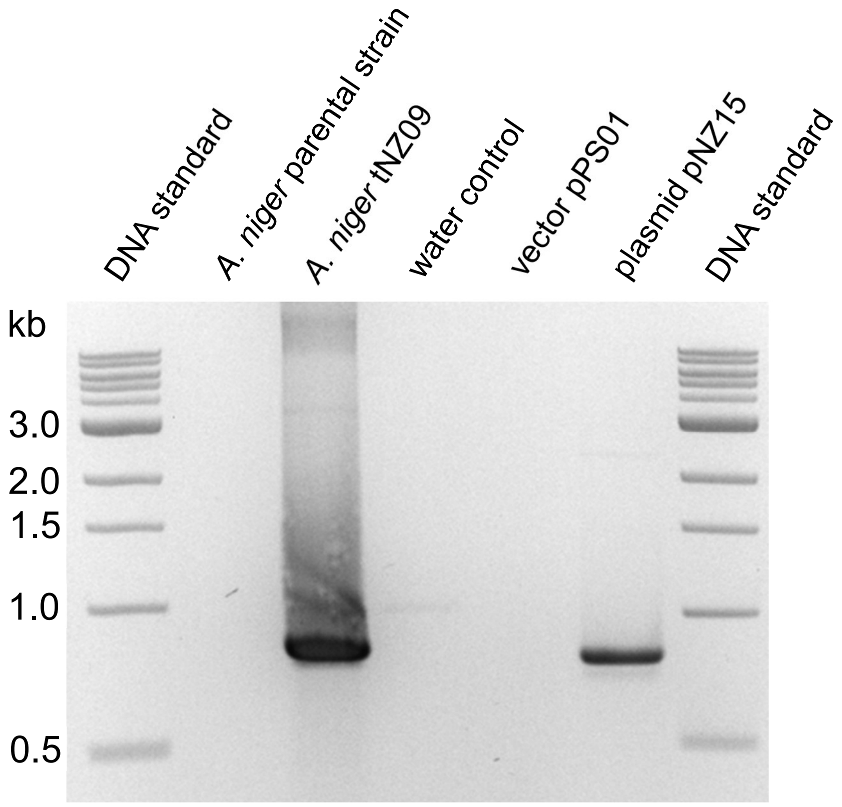

Figure S9. Agarose gel electrophoresis of PCRs to verify integration of the *cubC* expression construct pNZ15 in the *Aspergillus niger* genome. The integration yielded strain *A. niger* tNZ09. Negative controls i) with genomic DNA of the untransformed parental strain *A. niger* ATNT16ΔpyrGx24, ii) water control without template DNA, iii) a reaction with empty vector DNA (pPS01), and a positive control (plasmid pNZ15) are shown as well.

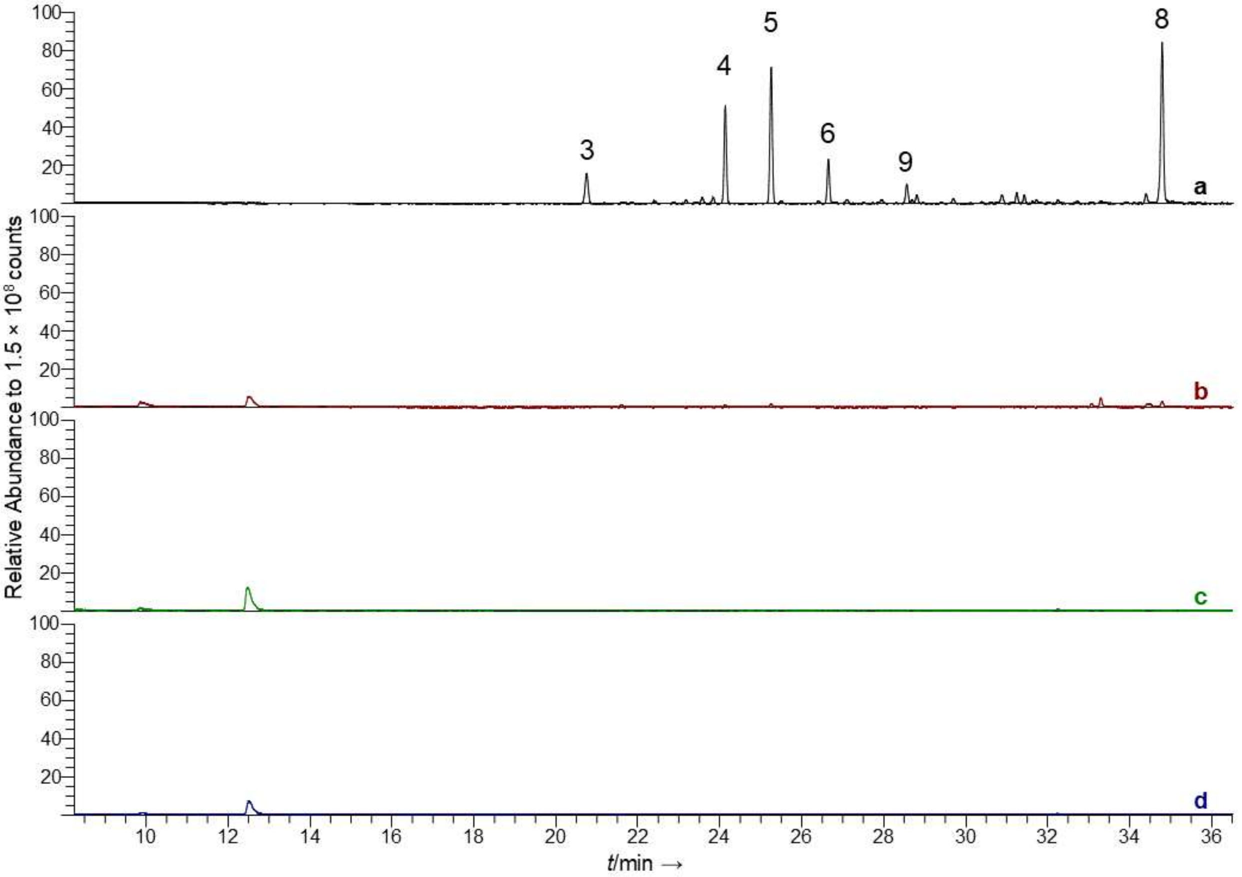

Figure S10. Gas chromatographic analysis of CubC-catalyzed terpenoid formation in *Aspergillus niger* tNZ09. Shown are *n*-hexane extracts of a (a) doxycycline-induced and (b) non-induced culture of *A. niger* tNZ09. Chromatograms c and d represent extracts of induced cultures of the empty vector control strain, *A. niger* tPS01, and the parental strain *A. niger* ATNT16ΔpyrGx24. Compound numbering: unidentified compound (**3**), β-elemene (**4**), β-caryophyllene (**5**), α-humulene (**6**), unidentified compounds (**8, 9**).

A

B

C

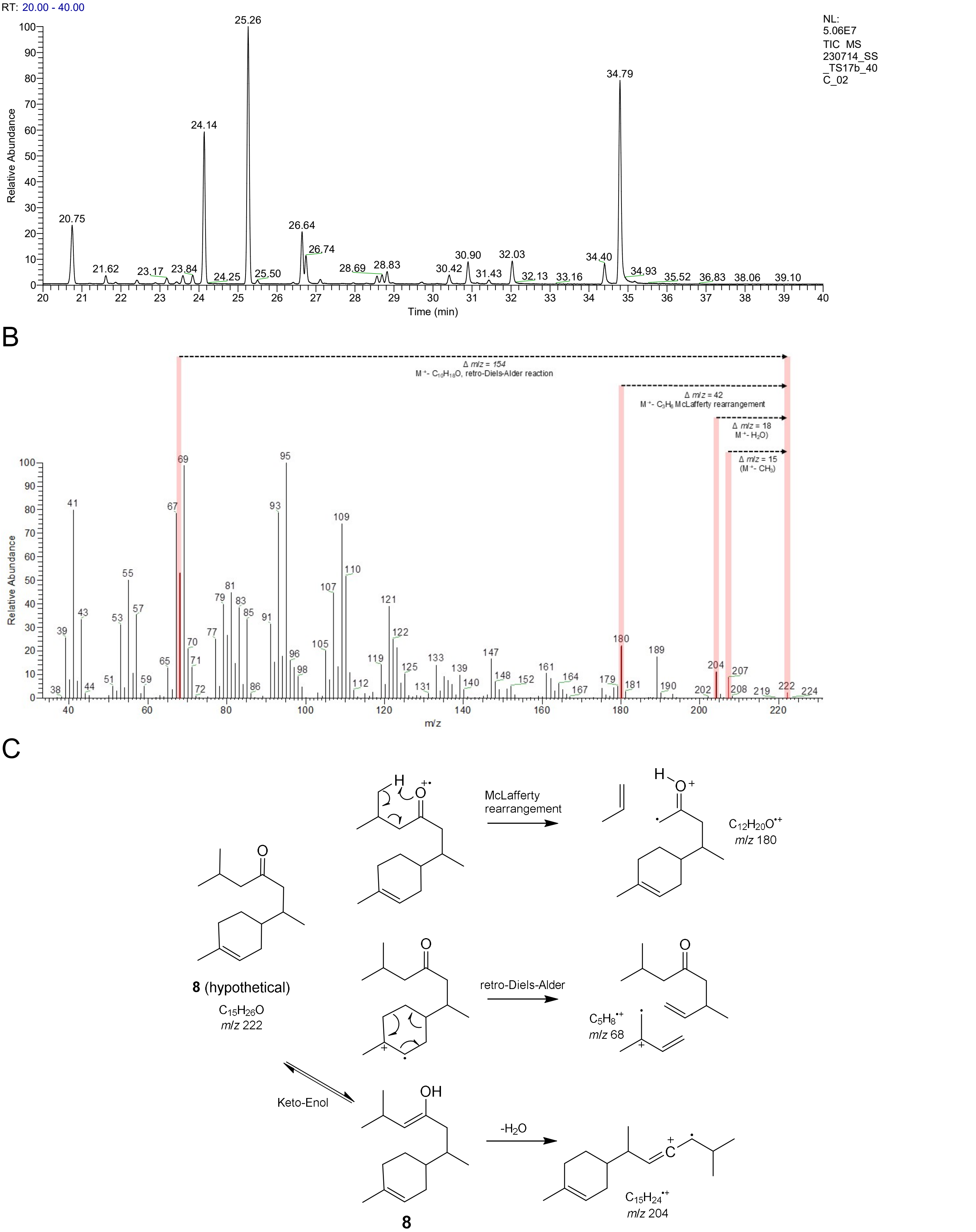

Figure S11. GC-MS chromatogram and MS spectrum of unidentified compound 8. Top panel: gas chromatographic separation of the CubC *in vitro* reaction with FPP as substrate. Bottom panel: Mass spectrum at t_R=34.79 min. Peaks of the molecular ion (*m*/*z* 222), cleavage of a methyl group (*m*/*z* 207), and the even fragments *m*/*z* 204 (loss of H_2O), *m*/*z* 180 (possible McLafferty rearrangement), and *m*/*z* 68 (retro-Diels-Alder reaction) are marked in red. Structures of the hypothetical **8** and the proposed fragments are shown below.

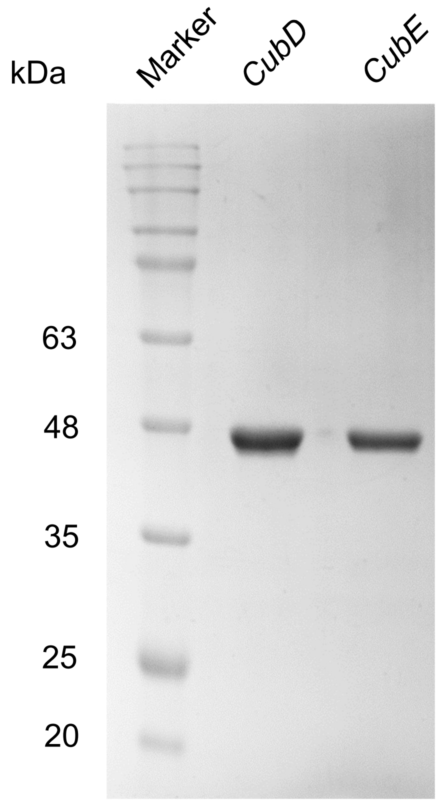

Figure S12. SDS polyacrylamide gel electrophoresis of purified His$_6$-tagged *Psilocybe cubensis* CubD and CubE. The calculated masses of the hexahistidine-tagged enzymes are 45.2 and 44.0 kDa, respectively. Protein standard: Blue Eye prestained marker (Jena Bioscience).

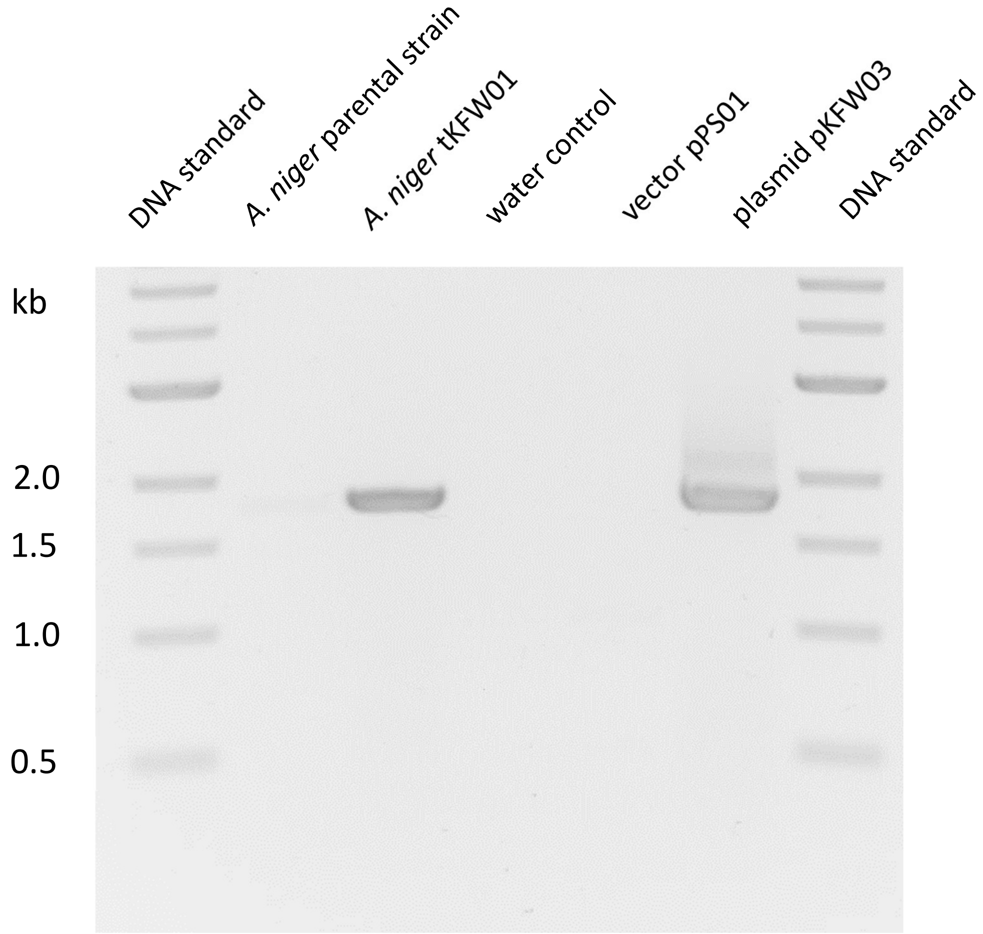

Figure S13. Agarose gel electrophoresis of PCRs to verify integration of the *cubD* expression construct pKFW03 in the genome of *Aspergillus niger*. The integration yielded strain *A. niger* tKFW01. Negative controls i) with genomic DNA of the untransformed parental strain *A. niger* ATNT16ΔpyrGx24, ii) water control without template DNA, iii) a reaction with empty vector DNA (pPS01), and a positive control (plasmid pKFW03) are shown as well.

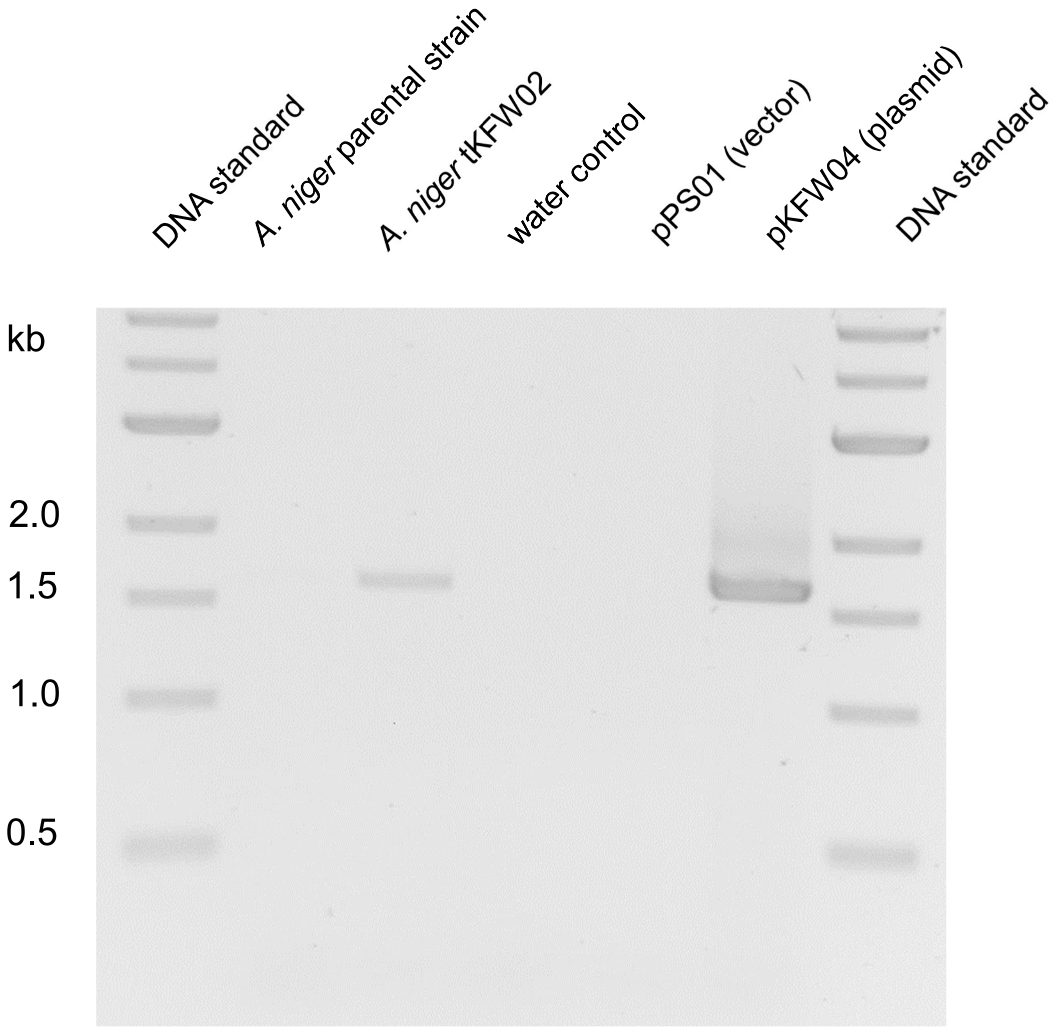

Figure S14. Agarose gel electrophoresis of PCRs to verify integration of the *cubE* expression construct pKFW04 in the genome of *Aspergillus niger*. The integration yielded strain *A. niger* tKFW02. Negative controls were i) genomic DNA of the untransformed parental strain *A. niger* ATNT16ΔpyrGx24, ii) a water control without template DNA, iii) a reaction with empty vector DNA (pPS01). The positive control was plasmid pKFW04 as PCR template.

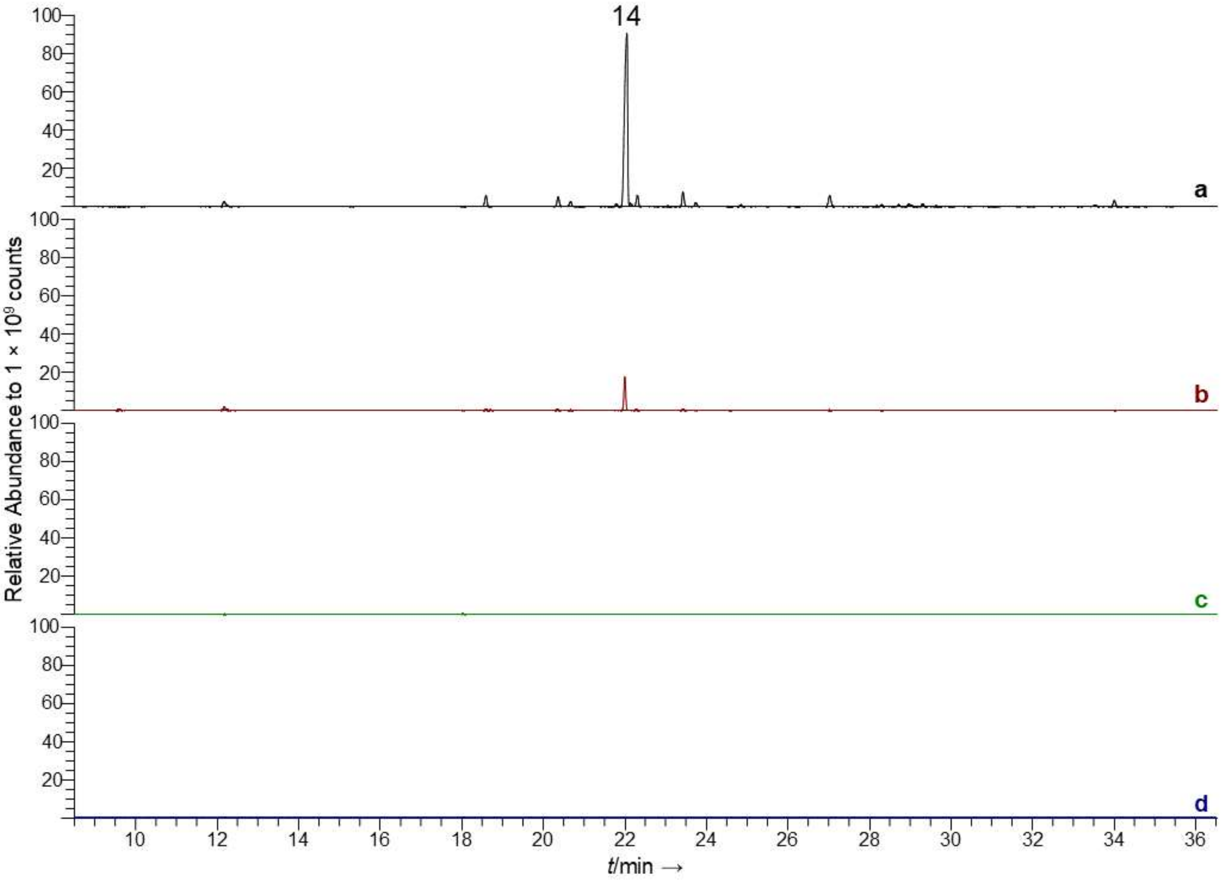

Figure S15. Gas chromatographic analysis of CubD-catalyzed terpenoid formation in *Aspergillus niger* tKFW01. Shown are *n*-hexane extracts of (a) doxycycline-induced and (b) non-induced culture of *A. niger* tKFW01. Chromatograms c and d represent extracts of induced cultures of the empty vector control strain, *A. niger* tPS01, and the induced parental strain *A. niger* ATNT16ΔpyrGx24. Compound numbering: sterpurene (**14**).

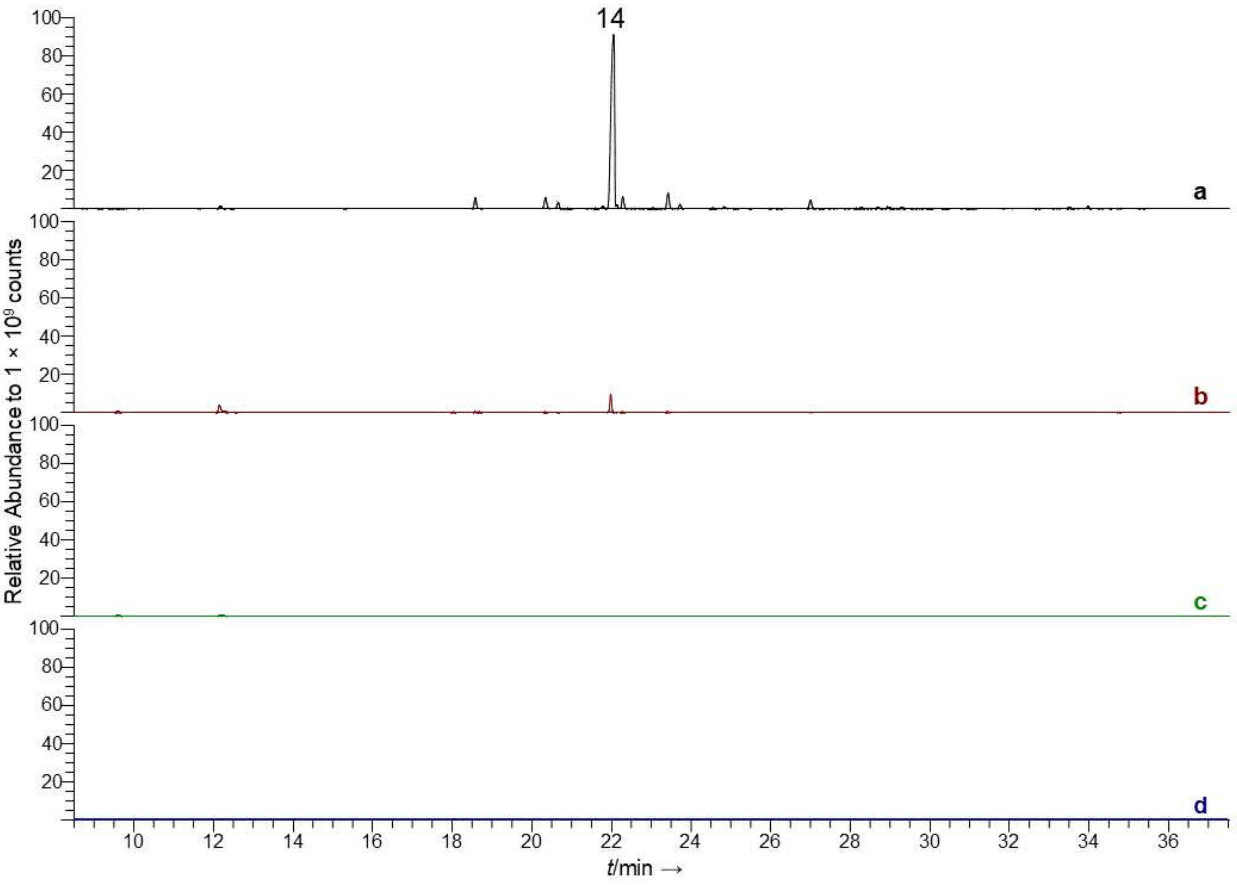

Figure S16. Gas chromatographic analysis of CubE-catalyzed terpenoid formation in *Aspergillus niger* tKFW02. Shown are *n*-hexane extracts of (a) doxycycline-induced and (b) non-induced culture of *A. niger* tKFW02. Chromatograms c and d show extracts of induced cultures of the empty vector control strain, *A. niger* tPS01, and the induced parental strain *A. niger* ATNT16ΔpyrGx24. Compound numbering: sterpurene (**14**).

Table S1. Representatives of clade III terpene synthases used for the phylogenetic analyses.

Clade	Protein	NCBI, PDB, or JGI ID, GenBank IDs for *cubB-cubE*	Organism	Sequence length (aa)	Reference (PUBMED ID)
clade III	Agr6	QGA30882.2	*Agrocybe/Cyclocybe aegerita*	345	32233445
	Agr7	A0A5Q0QNH9.1	*Agrocybe/Cyclocybe aegerita*	387	32233445
	Agr8	A0A5Q0QMX1.1	*Agrocybe/Cyclocybe aegerita*	353	32233445
	Agr9	A0A5Q0QMX0.1	*Agrocybe/Cyclocybe aegerita*	372	32233445
	Pro1	AGR34199.1	*Armillaria gallica*	345	21148562
	CpSTS1	LC436345.1	*Clitopilus pseudo-pinsitus*	362	31101615
	CpSTS4	LC436348.1	*Clitopilus pseudo-pinsitus*	414	31101615
	CpSTS6	LC436350.1	*Clitopilus pseudo-pinsitus*	360	31101615
	CpSTS7	BBH51504.1	*Clitopilus pseudo-pinsitus*	375	31101615
	Denbi1_659367	A0A4S8MAF3.1	*Dendrothele bispora*	353	32233445
	GlSTS6	UDP19923.1	*Ganoderma lucidum*	419	35252713
	GsSTS43	PIL26225.1	*Ganoderma sinense*	418	35252713
	GsSTS45b	UDP19925.1	*Ganoderma sinense*	410	35252713
	Hetan2_454193	XP_009550163.1	*Heterobasidion irregulare*	345	32233445
	Hfas94a	QDF59313.1	*Hypholoma fasciculare*	345	31392585
	Hfas94b	QDF59314.1	*Hypholoma fasciculare*	371	31392585
	Hypsu1_138665	A0A0D2L718.1	*Hypholoma sublateritium*	327	32233445
	LdSTS7	KAH9033225.1	*Lactarius deliciosus*	346	36594743
	LdSTS11	KAH9053987.1	*Lactarius deliciosus*	346	36594743
	Omp6	jgi|Ompol1|4774|MUStwsD_GLEAN_10003820	*Omphalotus olearius*	358	22726691
	Omp7	2271|MUStwsD_GLEAN_10000831	*Omphalotus olearius*	346	22726691
	PcubTS2	XP_047749409.1	*Psilocybe cubensis*	343	-
	PcubTS3 (CubD)	XP_047742792.1 (GenBank ID: PQ784406, *cubD*)	*Psilocybe cubensis*	364	this work
	PcubTS4a (CubB)	XP_047743958.1 (GenBank ID: PQ784404, *cubB*)	*Psilocybe cubensis*	344	this work
	PcubTS4b	XP_047743957.1	*Psilocybe cubensis*	344	-
	PcubTS6 (CubE)	XP_047744996.1 (GenBank ID: PQ784407, *cubE*)	*Psilocybe cubensis*	364	this work
	PcubTS10	XP_047744750.1	*Psilocybe cubensis*	371	-

	PcubTS17a	XP_047745566.1	*Psilocybe cubensis*	354	-
	PcubTS17b (CubC)	XP_047745564.1 (GenBank ID: PQ784405, *cubC*)	*Psilocybe cubensis*	361	this work
	PcubTS19	XP_047745687.1	*Psilocybe cubensis*	354	-
	PpSTS08	A0A348B784.1	*Postia placenta*	342	30105900
	PpSTS09	A0A348B785.1	*Postia placenta*	344	30105900
	PpSTS14	A0A348B788.1	*Postia placenta*	351	30105900
	ShSTS13	XP_007299393.1	*Stereum hirsutum*	315	24166732
	ShSTS15	XP_007308318.1	*Stereum hirsutum*	337	24166732
	ShSTS16	XP_007299456.1	*Stereum hirsutum*	369	24166732
	ShSTS18	P9WEW0.1	*Stereum hirsutum*	351	24166732
	SlacTC6	XP_007322499	*Serpula lacrymans var. lacrymans*	333	21764756

Table S2. Sequence similarities between *Psilocybe cubensis* CubB, CubC, CubD, and CubE, and predicted terpene synthases encoded by various *Psilocybe* species. Similarities for the putative terpene synthases PcubTS4b (encoded next to *cubB*) and PcubTS17a (encoded next to *cubC*) are shown as well.

Species	*cubB* homologs	identical/ similar aa (%)	PcubTS4b homologs	identical/ similar aa (%)	*cubC* homologs	identical/ similar aa (%)	PcubTS17a homologs	identical/ similar aa (%)	*cubD* homologs	identical/ similar aa (%)	*cubE* homologs	identical/ similar aa (%)
P. serbica	PserTS23a	89/96	PserTS23b	84/95	PserTS04b	61/80	PserTS04b	55/75	PserTS12	90/95	PserTS12	90/95
					PserTS12	61/81	PserTS12	55/74	PserTS4b	90/93	PserTS4b	90/93
					PserTS04a	59/80	PserTS04a	55/75				
					PserTS06	54/74	PserTS06	54/74				
P. mexicana	PmexTS15a	78/90	PmexTS15b	91/93	none		none		none		none	
P. cyanescens	PcyaTS07a	90/96	PcyaTS02	87/96	PcyaTS11	61/81	PcyaTS11	55/76	PcyaTS16	93/98	PcyaTS16	96/99
	PcyaTS08b	87/96	PcyaTS07b	85/96	PcyaTS16	61/82	PcyaTS16	55/75	PcyaTS11	90/94	PcyaTS11	90/95
	PcyaTS08a	85/92										
	PcyaTS09	85/91										
P. azurescens	PazuTS16a	90/97	PazuTS16c	87/95	PazuTS08	61/80	PazuTS08	55/75	PazuTS14	91/96	PazuTS14	94/98
	PazuTS18a	90/96	PazuTS18b	86/95	PazuTS14	60/81	PazuTS14	54/75	PazuTS08	88/92	PazuTS08	87/94
	PazuTS04a	90/96	PazuTS04b	85/96								
	PazuTS05a	88/96	PazuTS05b	73/84								

Table S3. Identified products in *in vitro* assays with *Psilocybe cubensis* CubB and (2*E*,6*E*)-FPP and (2*E*)-GPP, respectively, as substrate.

Compound	t_R (min)	Integral	Integral (% of total area)	Retention Index (observed)	Retention Index (Adams)[20a]	Retention Index (NIST)[20b]	Present in *Piper cubeba* oil?[36]	Present in *Elemi* oil?[37]
(3*R*,6*E*)-(-)-nerolidol (**1**, with FPP)	30.99	7.48×10^7	100	1565	1561	1564	yes	yes
(3*R*)-(-)-linalool (**2**, with GPP)	11.884	2.6×10^{10}	100	1100	1095	1099	yes	no

t_R and mass spectra identical with those of reference compound; absolute configuration confirmed by chiral GC.

Table S4. Identified product in extracts of *Aspergillus niger* tNZ07. Expression of *cubB* in this transgenic strain was induced with 30 µg mL^{-1} doxycycline.

Compound	t_R (min)	Integral	Integral (% of total area of monoterpenes)	Retention Index (observed)	Retention Index (Adams)[20a]	Retention Index (NIST)[20b]	Present in *Piper cubeba* oil?[36]	Present in *Elemi* oil?[37]
(3*R*)-(-)-linalool (**2**)	11.929	5.97×10^8	100	1100	1095	1099	yes	no

t_R and mass spectra identical with those of reference compound; absolute configuration confirmed by chiral GC.

Table S5. Sesquiterpenes identified after *in vitro* product formation assays with *Psilocybe cubensis* CubC and (2*E*,6*E*)-FPP as substrate. Percentages are referenced to the total integral of the indicated sesquiterpenoids (**3**-**8**). Indicated are compounds/signals with > 3% of the total areas under the curve. β-caryophyllene was additionally identified by chromatographic comparison with an authentic standard.

Compound	t_R (min)	Integral	Integral (% of total area)	Retention Index (observed)	Retention Index (Adams)[20a]	Retention Index (NIST)[20b]	Present in *Piper cubeba* oil?[36]	Present in *Elemi* oil?[37]
unidentified (**3**)	20.75	5.47×10^7	7.4	1312	-	-	-	-
β-Elemene (**4**)§	24.13	1.14×10^8	15.5	1393	1389	1391	yes	yes
β-Caryophyllene (**5**)	25.26	1.96×10^8	26.8	1421	1420	1419	no	no
α-Humulene (**6**)	26.64	4.00×10^7	5.4	1455	1452	1454	yes	no
β-Farnesene (**7**)	26.74	2.27×10^7	3.1	1457	1454	1457	yes	no
unidentified (**8**)	34.79	1.78×10^8	24.18	1666	-	-	-	-

§β-Elemene identified by MS in combination with RI and database search. All other additionally by comparison with pure reference compounds.

Table S6. Sesquiterpenes identified after heterologous production of CubC in *Aspergillus niger* tNZ09. Expression of *cubC* in this transgenic strain was induced with 30 µg mL^{-1} doxycycline. Percentages are referenced to the total integral of the selected compounds. Indicated are compounds/signals with > 3% of the total areas under the curve.

Compound	t_R (min)	Integral	Integral (% of total area of sesquiterpenes)	Retention Index (observed)	Retention Index (Adams)[20a]	Retention Index (NIST)[20b]	Present in *Piper cubeba* oil?[36]	Present in *Elemi* oil?[37]
unidentified (**3**)	20.74	1.16×10^8	5.83	1312	-	-	-	-
β-Elemene (**4**)§	24.13	3.14×10^8	15.8	1394	1389	1391	yes	yes
β-Caryophyllene (**5**)	25.26	4.34×10^8	21.8	1421	1420	1419	no	no
α-Humulene (**6**)	26.63	1.40×10^8	7.04	1455	1452	1454	yes	no
unidentified (**9**)	28.55	6.52×10^7	3.28	1503	-	-	-	-
unidentified (**8**)	34.78	5.75×10^8	28.91	1666	-	-	-	-

§β-Elemene identified by MS in combination with RI and database search. All other additionally by comparison with pure reference compounds.

Table S7. Monoterpenes and -terpenoids identified after *in vitro* product formation assays with *Psilocybe cubensis* CubC and (2*E*)-GPP as substrate. Percentages are referenced to total integral of the selected sesquiterpenoids. Indicated are compounds/signals with > 3% of the total areas under the curve.

Compound§	t_R (min)	Integral	Integral (% of total area)	Retention Index (observed)	Retention Index (Adams)[20a]	Retention Index (NIST)[20b]	Present in *Piper cubeba* oil?[36]	Present in *Elemi* oil?[37]
Myrcene (**10**)	8.01	1.21×10^8	3.7	991	988	991	yes	no
(*Z*)-β-Ocimene (**11**)	9.56	4.64×10^8	14.2	1036	1032	1037	no	no
(*E*)-β-Ocimene (**12**)	9.93	3.46×10^8	10.6	1046	1044	1049	yes	no
Linalool (**2**)	11.84	7.74×10^8	23.7	1100	1095	1099	yes	no
Geraniol (**13**)	18.2	1.56×10^9	47.8	1253	1249	1255	yes	no

§Compounds identified by MS in combination with RI and database search and by comparison with pure reference compounds.

Table S8. Sesquiterpenes identified after *in vitro* product formation assays with *Psilocybe cubensis* CubD and CubE and FPP as substrates.

CubD

Compound	t_R (min)	Integral	Integral (% of total area)	Retention Index (observed)	Retention Index (Massfinder)[20d]	Retention Index (NIST)[20b]	Present in *Piper cubeba* oil?[36]	Present in *Elemi* oil?[37]
Sterpurene (**14**)§	21.727	5.13×10^8	97.21	1350	1351	-	no	no

CubE

Compound	t_R (min)	Integral	Integral (% of total area)	Retention Index (observed)	Retention Index (Massfinder)[20d]	Retention Index (NIST)[20b]	Present in *Piper cubeba* oil?[36]	Present in *Elemi* oil?[37]
Sterpurene (**14**)§	21.724	6.65×10^8	95.29	1350	1351	-	no	no

§Identified by MS in combination with RI and Massfinder database[20d] search.

Table S9. Monoterpenes and -terpenoids identified after *in vitro* product formation assays with *Psilocybe cubensis* CubD and CubE. Assays were run with (2*E*)-GPP as substrate.

CubD

Compound§	t_R (min)	Integral	Integral (% of total area)	Retention Index (observed)	Retention Index (Adams)[20a]	Retention Index (NIST)[20b]	Present in *Piper cubeba* oil?[36]	Present in *Elemi* oil?[37]
Linalool (**2**)	11.306	1,55 × 10^8	27.71	1098	1095	1099	yes	no
para-Menth-2-en-1-ol (**15**)	12.888	4.88 × 10^7	8.72	1137	1136	-	no	no
α- Terpineol (**16**)	14.983	1.65 × 10^8	29.36	1189	1186	1189	yes	no
Geraniol (**13**)	17.639	1.75× 10^8	31.31	1253	1249	1255	yes	no

§Identified by MS in combination with RI and database search and, except of **16**, by comparison with pure reference compounds.

CubE

Compound	t_R (min)	Integral	Integral (% of total area)	Retention Index (observed)	Retention Index (Adams)[20a]	Retention Index (NIST)[20b]	Present in *Piper cubeba* oil?[36]	Present in *Elemi* oil?[37]
Linalool (**2**)	11.323	1.90 × 10^8	27.83	1098	1095	1099	yes	no
para-Menth-2-en-1-ol (**15**)	12.919	5.93 × 10^7	8.70	1138	1136	-	no	no
α- Terpineol (**16**)	14.997	2.02 × 10^8	29.70	1189	1186	1189	yes	no
Geraniol (**13**)	17.663	2.17 × 10^8	31.88	1253	1249	1255	yes	no

Identified by MS in combination with RI and database search and except of **16** by comparison with pure reference compounds.

Table S10. Sesquiterpenes identified after heterologous production of CubD in *Aspergillus niger* tKFW01 and of CubE in *A. niger* tKFW02. Expression of *cubD* and *cubE* was induced with 30 µg mL^{-1} doxycycline. Percentages are referenced to the total integral of the selected compounds. Indicated are compounds/signals with > 3% of the total areas of sesquiterpenes under the curve.

CubD

Compound	t_R (min)	Integral	Integral (% of total sequiterpen area)	Retention Index (observed)	Retention Index (Massfinder)[20d]	Retention Index (NIST)[20b]	Present in *Piper cubeba* oil?[36]	Present in *Elemi* oil?[37]
Sterpurene (**14**)	22.03	3.40×10^9	78.3	1352	1351	-	no	no
Unknown	23.42	1.51×10^8	3.48	1386	-	-	no	no
Unknown	27.01	1.39×10^8	3.20	1474	-	-	no	no

CubE

Compound	t_R (min)	Integral	Integral (% of total sesquiterpene area)	Retention Index (observed)	Retention Index (Massfinder)[20d]	Retention Index (NIST)[20b]	Present in *Piper cubeba* oil?[36]	Present in *Elemi* oil?[37]
Unknown	20.34	2.49×10^8	3.03	1312	-	-	no	no
Sterpurene (**14**)	22.05	6.01×10^9	74.39	1353	1351	-	no	no
Unknown	23.41	3.42×10^8	4.17	1385	-	-	no	no

Identified by MS in combination with RI and Massfinder database[20d] search.

Table S11. Hydrodestillation of *Psilocybe cubensis* biomass. Sesquiterpenes with peak areas below 1% of the total area under the curves are not listed. Compounds detected after distillation of vegetative mycelium.

Compound	t_R (min)	Integral	Integral (% of total area of Sesquiterpenes)	Retention Index (observed)	Retention Index (Database)[20a,d]	Retention Index (NIST)[20b]	Present in *Piper cubeba* oil?[36]	Present in *Elemi* oil?[37]	Synthesizing Enzyme
Sterpurene (**14**)	21.90	2.0×10^9	5.19	1350	1351§	-	no	no	CubD, CubE
Protoillud-6-ene	23.00	2.0×10^9	5.07	1377	1382§	-	no	no	?
β-Elemene (**4**)	23.65	5.5×10^8	1.43	1392	1389	1391	yes	yes	CubA[34] CubC
β-Caryophyllene (**5**)	24.71	1.7×10^9	4.47	1418	1420	1419	yes	no	CubC
β-Copaene	25.25	1.4×10^9	3.52	1431	1430	1432	yes	no	CubA[34]
α-Muurolene	28.04	1.1×10^{10}	28.11	1501	1500	1499	yes	no	?
δ-Cadinene	28.94	1.2×10^9	3.16	1524	1522	1522	yes	no	CubA[34]
Nerolidol (**1**)	30.49	1.4×10^9	3.66	1564	1561	1564	yes	no	CubB
1-epi-Cubenol	32.95	7.8×10^8	2.01	1628	1627	1627	yes	no	?
Cubenol	33.48	1.2×10^9	3.05	1643	1645	1642	yes	no	?
α-Muurolol	33.64	1.4×10^{10}	35.56	1647	1644	1645	yes	no	?

§ Massfinder library,[20d] all others: Adams library[20a]

Compounds detected after distillation of fruiting bodies.

Compound	t_R (min)	Integral	Integral (% of total area of Sesquiterpenes)	Retention Index (observed)	Retention Index (Database)[20a]	Retention Index (NIST)[20b]	Present in *Piper cubeba* oil?[36]	Present in *Elemi* oil?[37]	Synthesizing Enzyme
Geranyl acetone	26.07	4.6×10^7	14.0	1453	1453	1453	no	no	?
Cubebol	28.62	7.3×10^6	2.2	1516	1514	1515	yes	no	CubA[34]
Nerolidol (**1**)	30.48	1.2×10^8	37.2	1564	1561	1564	yes	no	CubB
α-Muurolol (**18**)	33.61	1.1×10^7	3.3	1647	1644	1645	yes	no	?
(*E*,*E*)-Farnesol	36.39	1.4×10^8	43.2	1723	1722	1722	yes	no	?

Table S12. Oligonucleotides used for qRT-PCR. Primer efficiency, the linear correlation coefficient (R^2) and the expected amplicon size are indicated for the respective primer pairs.

Name	Sequence (5´→ 3´)	Target	Efficiency (R^2)	Amplicon size (bp) gDNA	Amplicon size (bp) cDNA
oMG388	GTGTCAACAACAACATCATTCC	*gpdA*	89% (0.9999)	208	133
oMG389	AGATCAACGACAGAGACATCG				
oMG386	CGAGAAGAGCTACGAACTGC	*actA*	92% (0.99994)	173	123
oMG387	GTCTCGTGGATACCGACGG				
oMG433	GTGGTGCCCAGGTGATGATC	*mtdA*	92% (0.99979)	172	115
oMG434	CAATAATCAACATTGCCCACACTG				
oNZ33	CAATGATGGAAGGGGCAACG	*cubB*	93% (0.99935)	159	103
oNZ34	GCGGTGATAACGTTGTGACCA				
oNZ41	ACAAAAGAAACTTCCCAACCTACG	*cubC*	94% (0.99994)	201	145
oNZ42	TGTATTTCCAGCCCTTCGTCC				
oKFW14	CCGCTAGCATTTTAACTACCC	*cubD*	94% (0.99884)	83	83
oKFW15	TGGTTCCGCGGATTCTTTTTTC				
oKFW16	GCTTCTACCGAGCCTAAAGTTC	*cubE*	89% (0.99715)	89	89
oKFW17	AAAGTATCGGGAAGCATG				

Table S13. Oligonucleotides used to construct *Escherichia coli* expression plasmids. Recognition sites for restriction enzymes *Bam*HI (orange), *Nhe*I (red), *Not*I (blue), *Xho*I (black) are shown in bold.

Name	Sequence (5´→ 3´)	Target (cDNA)
oNZ15	GCGGCAGCCATATG**GCTAGC**ATGTCTTCCACTGAATTCGTGATC	*cubB*
oNZ16	TGGTGGTGGTGGTG**CTCGAG**TTACGCTAAGCTGCTTTGAAG	
oNZ23	GCGGCAGCCATATG**GCTAGC**ATGTCAACAGTGAACATCACC	*cubC*
oNZ24	TGGTGGTGGTGGTG**CTCGAG**TTAAGCAATGACAACTTTACGAG	
oNZ13	AGCAAATGGGTCGC**GGATCC**ATGTCCACCGCTAGCATTTTAAC	*cubD*
oNZ14	TGGTGGTGGTGGTG**CTCGAG**TTATGCAATGATGACTTTGCGAG	
oNZ17	GCGGCAGCCATATG**GCTAGC**ATGTCCACCGCTAACGTTGC	*cubE*
oNZ18	GAAAACTCGCAAAGTTATCATTGCTTAA**GCGGCCGC**A**CTCGAG**CACCA	

Table S14. Oligonucleotides used to construct *Aspergillus niger* expression plasmids. Recognition sites for restriction enzymes for *SpeI* are are shown in bold, for *PacI* sites are highlighed in red.

Name	Sequence (5´→ 3´)	Target (cDNA)
oNZ11	ATCACAGCACCATG**ACTAGT**ATGTCAACAGTGAACATCACCTC	*cubB*
oNZ12	CACTGCTGCTAG**TTAATTAA**TCAAGCAATGACAACTTTACGAG	
oNZ03	ATCACAGCACCATG**ACTAGT**ATGTCTTCCACTGAATTCGTGATCC	*cubC*
oNZ04	CACTGCTGCTAG**TTAATTAA**CTACGCTAAGCTGCTTTGAAG	
oKFW06	TTCTCATCACAGCACCATG**ACTAGT**ATGTCCACCGCTAGCATTTTAAC	*cubD*
oKFW07	GAAATCACTGCTGCTAG**TTAATTAA**TTATGCAATGATGACTTTGCGAG	
oKFW09	TTCTCATCACAGCACCATG**ACTAGT**ATGTCCACCGCTAACGTTGC	*cubE*
oKFW10	GAAATCACTGCTGCTAG**TTAATTAA**TTAAGCAATGATAACTTTGCGAGTTTTC	

Table S15. Oligonucleotides used for diagnostic PCR to verify transgene integration.

Name	Sequence (5´→ 3´)	Target
oMG360	CCTCCAAGAGAGATCCAGAC	vector pPS01
oMG370	GATCCTCTCTCTGATATTGTCG	vector pPS01
oNZ26	GCCCCTTCCATCATTGAAATTACC	*cubB*
oNZ30	CAATGGTGTCTCTGTTAAGGCG	*cubC*
oKFW08	GTGACCGCGAATCCATTGG	*cubD*
oKFW11	CGACACCAACTTTGTTATTGTGC	*cubE*

References

[4a] S. Dörner, K. Rogge, J. Fricke, T. Schäfer, J. M. Wurlitzer, M. Gressler, D. N. K. Pham, D. R. Manke, A. R. Chadeayne, D. Hoffmeister, *ChemBioChem* **2022**, *23*, e202200249.

[17] a) J. Fricke, F. Blei, D. Hoffmeister, *Angew. Chem. Int. Ed.* **2017**, *56*, 12352–12355, *Angew. Chem*. 2017, *129*, 12524–12527; b) H. T. Reynolds, V. Vijayakumar, E. Gluck-Thaler, H. B. Korotkin, P. B. Matheny, J. C. Slot, *Evol. Lett*. **2018**, *2*, 88–101.

[20] a) R. P. Adams, Identification of Essential Oil Components by Gas Chromatography/Mass Spectrometry, Allured Publishing Corp., Carol Stream, IL, **2007**; b) National Institute for Standards and Technology, Mass Spectral & Retention Index Library, **2017**; d) Massfinder. Massfinder. 4.21 ed. Hamburg, Germany: Hochmuth Scientific Consulting; **2010** (MS/RI-library, mainly based on [20c] with additional compounds).

[32] K. B. Lengeler, E. Kothe, *Curr. Genet*. **1999**, 36, 159–164.

[33] R. Demmler, J. Fricke, S. Dörner, M. Gressler, D. Hoffmeister, *ChemBioChem* **2020**, 21,1364–1371.

[34] E. Schäfer, P. S. Seibold, S. Bartram, F. Trottmann, V. G. Haensch, M. Gressler, A. R. Chadeayne, C. Hertweck, S. E. O'Connor, D. Hoffmeister, *ChemBioChem* **2023**, *24*:e202300511.

[36] a) R. Bos, H. J. Woerdenbag, O. Kayser, W. J. Quax, K. Ruslan, Elfami, *J. Essent. Oil Res*. **2007**, 19, 14–17; b) J. C. Chalchat, R. P. Garry, *J. Essent. Oil Res*. **1997**, 9, 311−319.

[37] M. A. Villanueva, R. C. Torres, K. H. C. Baser, T. Özek, M. Kürkçüoglu M, *Flavour Frag. J*. **1993**, *8*, 35−37.

[38] A. J. Bradshaw, V. Ramírez-Cruz, A. R. Awan, G. Furci, L. Guzmán-Dávalos, B. T. Dentinger, *Proc. Natl. Acad. Sci. USA* **2024**, *121*: e2311245121.

[39] K. McKernan, L. T. Kane, S. Crawford, C.-S. Chin, A. Trippe, S. McLaughlin, *F1000Res*, **2021**,*10*:281.
